# Fabrication of nitrogen-hyperdoped silicon by high-pressure gas immersion excimer laser doping

**DOI:** 10.1038/s41598-024-69552-8

**Published:** 2024-08-23

**Authors:** Josh W. Barkby, Fabrizio Moro, Michele Perego, Fabiana Taglietti, Elefterios Lidorikis, Nikolaos Kalfagiannis, Demosthenes C. Koutsogeorgis, Marco Fanciulli

**Affiliations:** 1 Department of Physics and Mathematics, Nottingham Trent University, Nottingham, NG11 8NS UK; 2 Dipartimento di Scienza dei Materiali, Università degli Studi di Milano-Bicocca, 20125 Milan, Italy; 3 Materials and Devices for Microelectronics (MDM) Laboratory, Institute for Microelectronics and Microsystems (IMM), National Research Council (CNR), Via C. Olivetti 2, 20864 Agrate Brianza, MB Italy; 4 Department of Materials Science and Engineering, University of Ioannina, 45110 Ioannina, Greece

**Keywords:** Materials science, Physics

## Abstract

In recent years, research on hyperdoped semiconductors has accelerated, displaying dopant concentrations far exceeding solubility limits to surpass the limitations of conventionally doped materials. Nitrogen defects in silicon have been extensively investigated for their unique characteristics compared to other pnictogen dopants. However, previous practical investigations have encountered challenges in achieving high nitrogen defect concentrations due to the low solubility and diffusivity of nitrogen in silicon, and the necessary non-equilibrium techniques, such as ion implantation, resulting in crystal damage and amorphisation. In this study, we present a single-step technique called high-pressure gas immersion excimer laser doping (HP-GIELD) to manufacture nitrogen-hyperdoped silicon. Our approach offers ultrafast processing, scalability, high control, and reproducibility. Employing HP-GIELD, we achieved nitrogen concentrations exceeding 6 at% (3.01 × 10^21^ at/cm^3^) in intrinsic silicon. Notably, nitrogen concentration remained above the liquid solubility limit to ~1 µm in depth. HP-GIELD’s high-pressure environment effectively suppressed physical surface damage and the generation of silicon dangling bonds, while the well-known effects of pulsed laser annealing (PLA) preserved crystallinity. Additionally, we conducted a theoretical analysis of light-matter interactions and thermal effects governing nitrogen diffusion during HP-GIELD, which provided insights into the doping mechanism. Leveraging excimer lasers, our method is well-suited for integration into high-volume semiconductor manufacturing, particularly front-end-of-line processes.

## Introduction

Over the last decade, there has been a surge in research focused on hyperdoped semiconductors, aiming to surpass the limitations of conventionally doped materials by achieving dopant concentrations well beyond the solubility limits^1^. Nitrogen (N)-hyperdoped silicon (Si) has emerged as a promising material with desirable electronic, spintronic, and photonic properties, offering a wide range of potential applications. N defects in Si exhibit strong sub-bandgap infrared (IR) local vibrational mode (LVM) absorptions^2–9^ suitable for enhancing optoelectronics, photovoltaics, and detectors^10,11^. N also enhances oxygen precipitation and interacts significantly with vacancies, altering void size and kinetics and suppressing intrinsic defect formation^12–16^. Many N defect structures can exist within the Si crystal with a wide range of formation energies. Some examples from Zhu et al., 2015^17^ are given in Table 1. The substitutional N defect in Si (N_*Si*_) is a deep donor^18,19^, enabling observation of the unpaired electron spin by electron paramagnetic resonance (EPR) at room temperature (RT) and above^20^. With reliable fabrication, exploiting N_*Si*_ defects has the potential to advance quantum information processing by enabling high-temperature-operating devices^21,22^. In contrast to other shallow donor pnictogens, like phosphorous^23^, which are classically engineered through time-intensive multi-stage processes such as thermal diffusion, the fabrication of hyperdoped materials requires non-equilibrium techniques that go beyond traditional approaches to overcome the solubility limits and minimise post-doping thermal annealing (TA). However, previous attempts to dope Si with N^24,25^ using non-equilibrium techniques (e.g., ion implantation) have encountered difficulties achieving high N concentrations due to the very low solid and liquid solubility limits of only 4*.*5 × 10^15^ and 6*.*0 × 10^18^ at/cm^3^, respectively^26^, as well as N’s limited diffusivity in Si melt of 1*.*4 × 10^−5^ cm^2^ s^*−*1^ ^27^, and faced challenges due to crystal damage and amorphisation, requiring TA for recrystallisation. Additionally, TA fails to activate interstitial N into substitutional sites^28,29^, with the dominant and most stable N defect in Si being N_*i*_–N_*i*_^5,30^. It is only after laser annealing that N_*Si*_ defects are formed^20,29,31^. Previous laser irradiation studies have achieved the highest measured total N concentration in Si to date, of 0.5 ± 0.2 at% (2.5 × 10^20^ at/cm^3^)^32^. A well-understood non-equilibrium laser fabrication method is crucial to produce N-hyperdoped Si with significant N_*Si*_ concentrations. Thus, the motivation behind this work was to show that processing with high-energy excimer lasers can produce N-hyperdoped Si, and more specifically maximise the activated N defects into substitutional sites, introduced efficiently in a single step (introduction and activation in one processing step, rather than doping via a first implantation step and activation via a second annealing step). The novelty of a single step offers facile implementation, rapid application and reproducibility.

**Table 1:** Formation energies per N atom of N defect structures in crystalline silicon^17^.

Defect	Symbol	Formation energy (eV)
Di-interstitial pair	N_*i*_–N_*i*_	0
Humble ring structure	N/a	0.94
Single interstitial and single substitutional	N_*i*_–N_*s*_	1.13
Two substitutional N and a vacancy	N_*Si*_–N_*Si*_–V	1.52
Single split interstitial	N_*i*_	1.89
Single substitutional N, off-centre	N_*Si*_	2.10
Single substitutional N, on-centre	N_*Si*_	2.15
Bond-bridge	N_*b*_	2.19
N at a hexagonal site	N_*h*_	4.26
N at a tetrahedron site	N_*t*_	4.89

## Methods

**Figure 1:**
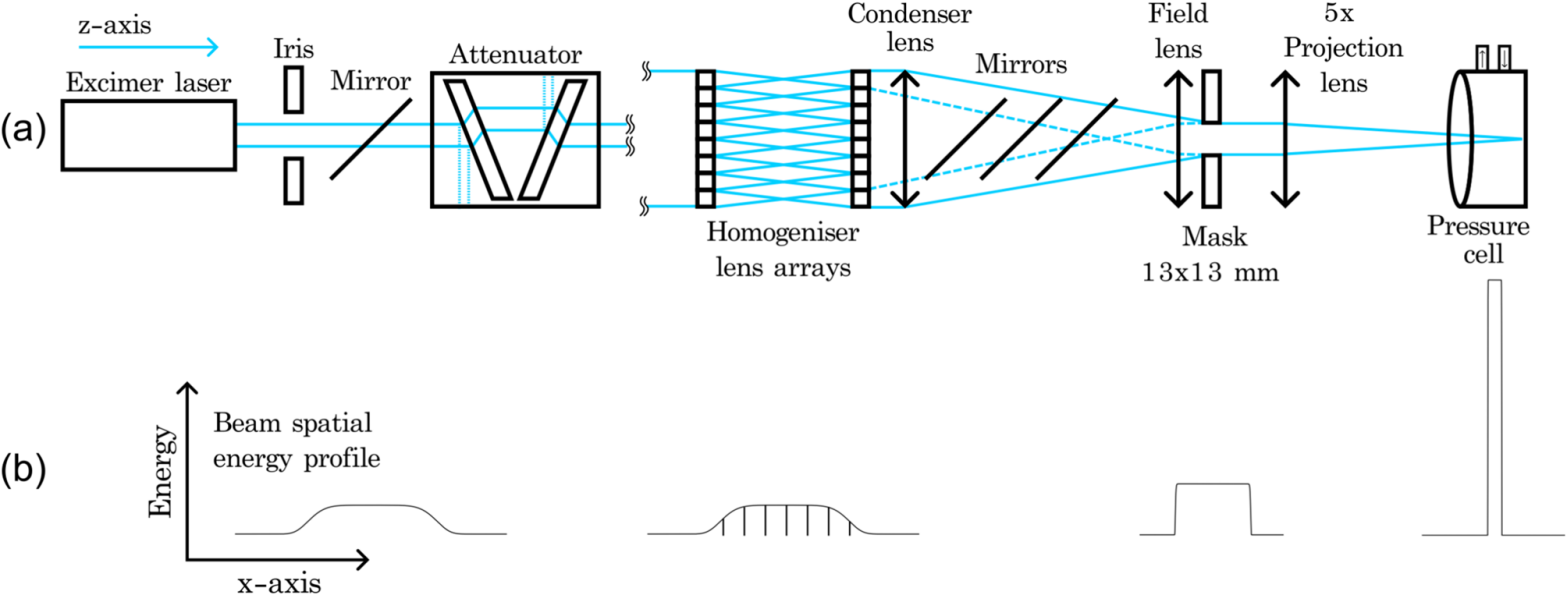
(**a**) Laser beam delivery diagram. (**b**) Beam spatial energy profile displaying the integration homogenisation of the initially Gaussian beam to a top-hat or “super-Gaussian”.

Intrinsic silicon (i-Si) wafers (prime grade, float-zone, 100 ± 0.3 mm diameter, 525 ± 25 µm thick, (100) ± 0.5° orientation, > 1000 Ω cm resistivity, < 10 µm total thickness variation, < 30 µm bow warp, single side polished) from Sil’tronix Silicon Technologies were first diced using a Trumpf TruMark 3230 frequency-doubled 532 nm Nd:YVO_4_ laser with a galvanometer beam deflection system and F-Theta lens to maintain constant focus across the processing plane. Full details of the wafer dicing process can be found in the supplementary information (Figs. S1, S2 and S3). Die were mounted to a carrier wafer with double-sided tape (shadowed by the die) and fixed into a recessed motorised XYZ stage. A fused silica window, measured by transmission spectrometry (Fig. S4) to have a 248 nm transmittance > 90% (Fig. S5), was mounted to form a 500 cm^3^ pressure cell, in which die are processed. For this work, the cell was pressurised with either argon (Ar), nitrogen (N_2_), or anhydrous ammonia (NH_3_), following multiple fill-vent cycles to ensure > 99.99% precursor gas environment purity. Transmission spectrometry of the gasses used in this study (Fig. S6) showed all precursors had a 248 nm transmittance of 98 ± 2% compared to atmosphere (Fig. S7). Samples were processed with a Lambda Physik LPX 305i excimer laser filled with a krypton fluoride (KrF) gas mix, measured by a fibre-coupled CCD spectrometer to lase at 248.5 ± 0.4 nm (Fig. S8), equating to a photon energy of ≈5 eV, sufficient to overcome the formation energies of nitrogen defects in silicon^17,30^, but not to break the molecular nitrogen (${\text{N}} \equiv {\text{N}}$) bond of 9.7985 eV^33^. Overcoming the ${\text{N}} \equiv {\text{N}}$ bond was achieved via other unique laser-matter interaction phenomena, discussed later. The laser was fired at a pulse repetition frequency of 1 Hz. Temporal intensity profile measurements using a fast PIN photodiode and oscilloscope quantified a full width at half maximum (FWHM) pulse duration of 42 ± 2 ns (Fig. S9). The energy output was tuned via a high-voltage discharge thyratron (14–23 kV), with the maximum energy per pulse measured to be 1400 ± 30 mJ. Beam delivery (Fig. 1) comprised a two-plate compensated variable attenuator which provided very fine fluence control. An integrator homogeniser system consisting of two 8 × 8 lens arrays followed by a condenser lens achieved a top-hat (high-order “super-Gaussian”) spatial intensity profile with a low uniformity variance (≈4.5%) across the maximum intensity, without hot spots. A 13 × 13 mm aperture metal shadow mask was positioned at the focal point of the homogeniser on a mask projection carousel, with a field lens just prior which compensated for divergence and provided full overlap of beamlet images at the projection lens that focused the beamlets at the sample surface^34^ with FWHM dimensions of 2.5 ± 0.1 mm^2^, measured by a Gentec-EO Beamage-4 M CCD spatial intensity profiler with a BSF23G11.3N UV–Vis fluorescent crystal converter (Fig. S10). The surface of the die were processed with various fluences and number of pulses in a grid of laser spots, overlapping one another and the edges of the die by 0.1 mm which ensured complete processing of the Si surface. Stage motion and laser firing were digitally controlled by a G-code program.

**Figure 2:**
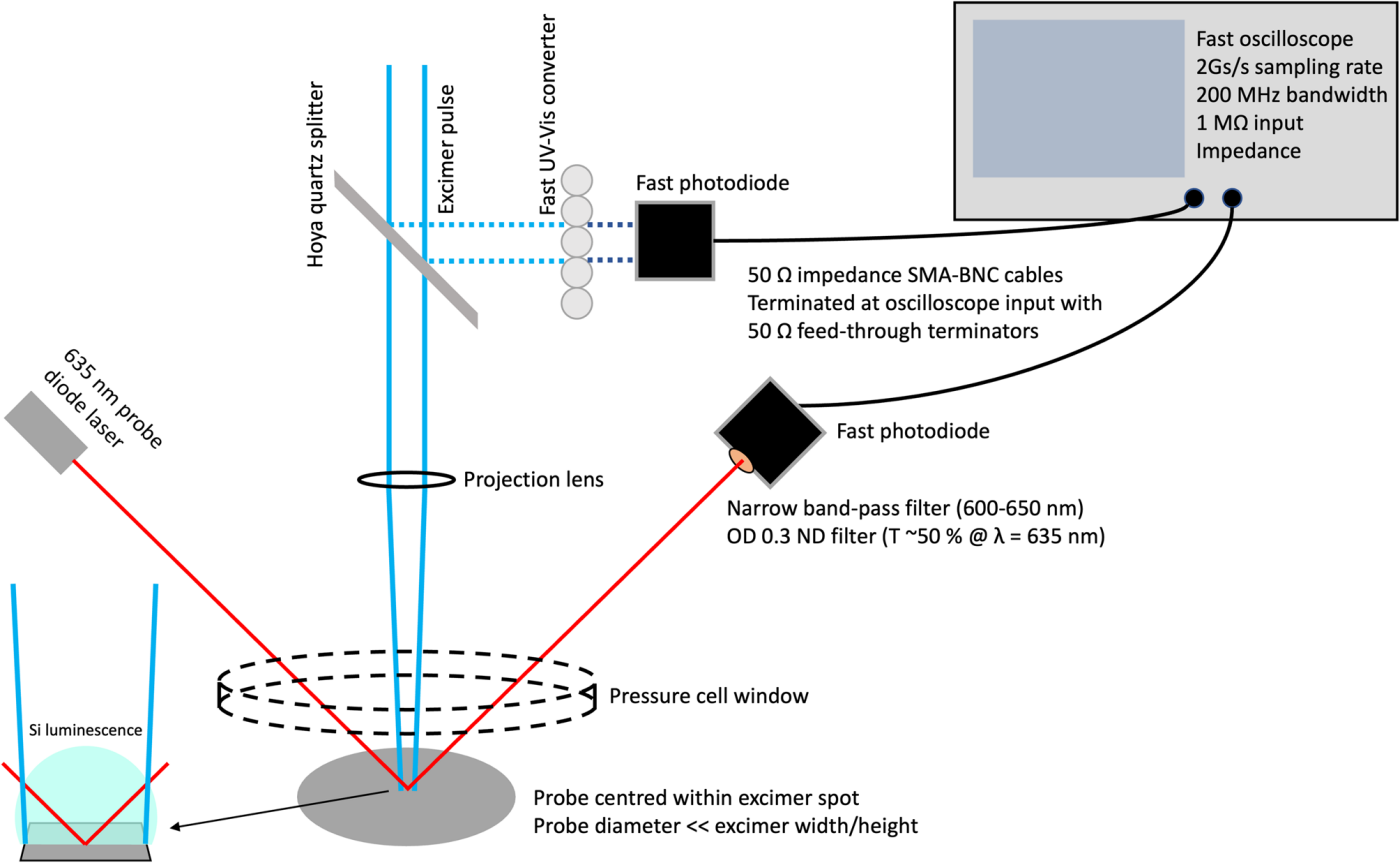
Schematic of the set-up used to simultaneously measure the temporal intensity profile of the excimer pulse and time-resolved reflectivity of the silicon surface during irradiation. Oscilloscope triggering was synced to the rising edge of the excimer pulse.

To gain insights into the laser annealing process of i-Si, we employed a specialised simulation tool for optothermal modelling (Full method detailed in the supplementary information). This tool helped enhance our understanding of the phenomena that occur during laser interactions with Si, particularly in the context of the HP-GIELD processes. Specifically, we employed strongly coupled optical and thermal simulations considering the corresponding properties of Si and their temperature-dependent behaviour. In particular, we utilised a 1D optical transfer matrix calculation and 1D time evolution of heat and combine the two into a coupled self-consistent module that contains all the material properties one would require, including optical dispersion and thermal properties (heat capacity, thermal conductivity (Figs. S13, S14, respectively)) that are temperature dependent, and phase transformation of materials (i.e., explicitly include melting point and latent heat of phase transformation). All computations were performed on a supercomputer CPU cluster (VIKOS) at the Department of Materials Science and Engineering at the University of Ioannina, Greece. Absorption was calculated at specific points in Si, based on the 5 nm grid step discretization we define, upon which the heat transport simulation is also performed. Coupled with the spectral intensity profile of the laser (Fig. S8) we get a spectral absorption profile, which, when integrated over all wavelengths, yielded the total absorption distribution per unit power incident. Finally, we coupled this with the temporal envelope of the laser provides the distributed time-dependent heating source used in the heat diffusion simulations. To experimentally explore the HP-GIELD process, simultaneous temporal intensity profile measurements of the excimer pulse and time-resolved reflectivity (TRR) measurements of the Si surface were performed during laser irradiation. A MenloSystems FPD510-FV PIN fast photodiode (2 ns rise time) with a 200 MHz bandwidth (− 3 dB) was used to sample the excimer beam, a 635 nm continuous wave (CW) AlGaInP diode laser (< 5 mW) to probe the silicon, and a Thorlabs DET025A/M PIN fast photodiode (150 ps rise time) with a 2 GHz bandwidth (− 3 dB) to sample the probe beam. Both photodiodes were connected via 50-Ω feed-through terminators to a GWINSTEK GDS-2202A fast oscilloscope sampling at 2 Gs/s (Fig. 2). A Hoya quartz plate was used as a beam splitter to reflect ~4% of the incident excimer pulse at each interface (≈7.84% total). Bubble wrap was used to convert the UV light to visible without persistent luminescence, avoiding temporal elongation of the pulse. An Ealing 622AF45 narrow band-pass filter (600–650 nm) and a Thorlabs NDUV503B neutral density filter with an optical density of 0.3 (transmission ≈50% at λ = 635 nm) were mounted to the photodiode collecting the probe reflection to eliminate the unwanted auxiliary photonic effects of room lighting, excimer pulse, or Si luminescence, and only collect the probe signal encoded with the temperature-dependent reflectivity of silicon, *R*_*Si*_(*T*). The intensity of the reflected probe beam, *I*_*refl*_, is the product of the emitted probe intensity, *I*_*probe*_, and *R*_*Si*_(*T*). We confirmed the photodiode output voltage signal, *V*_*pd*_, has a linear response to incident intensity (Fig. S15). To correlate *V*_*pd*_ to *R*_*Si*_, we followed the methodology of Diez et al.^35^ and solved Equation S11 for *R*_*Si*_ (see supplementary information in section ‘TRR’).

Dual-beam depth profiling time-of-flight secondary ion mass spectrometry (ToF-SIMS) measurements were performed using an IONTOF TOF-SIMS IV system to quantify total nitrogen concentration as a function of depth. A Cs^+^ sputtering beam was scanned over a 300 × 300 µm^2^ area at 1 keV for depth profiling. A Ga^+^ primary beam was scanned for analysis over the central 50 × 50 µm^2^ area of the sputtering crater at 25 keV. The analyser was used in negative polarity with a cycle time of 100 µs and mass range of 0–80% to detect N’s negative secondary ions. The minimum N concentration sensitivity of the system is 10^17^ at cm^−3^. The initial transient region (TR, before the ^30^Si matrix signal had stabilised) of ≈ 20 nm was excluded from the analysis. Detection of unpaired electron spins due to defects introduced in the fabricated materials was performed by continuous wave (CW) electron paramagnetic resonance (EPR). Measurements were performed with a Varian E15 EPR spectrometer equipped with a Bruker EF4122sHQ super high Q cavity and operated at X-band microwave frequencies (*ν *≈ 9.3 GHz) in absorption mode. The static magnetic field was modulated at 100 kHz. The microwave frequency and magnetic field, *B*, were monitored continuously with an electronic frequency counter and a Hall probe, respectively. The magnetic field was calibrated using a 2,2-diphenyl-1-picrylhydrazyl (DPPH, *g* = 2*.*0036(3)) standard. CW EPR spectra simulations and fittings were performed using the “pepper” function of EasySpin 5.2.35, an open-source MATLAB toolbox^36^.

## Results and discussion

**Figure 3:**
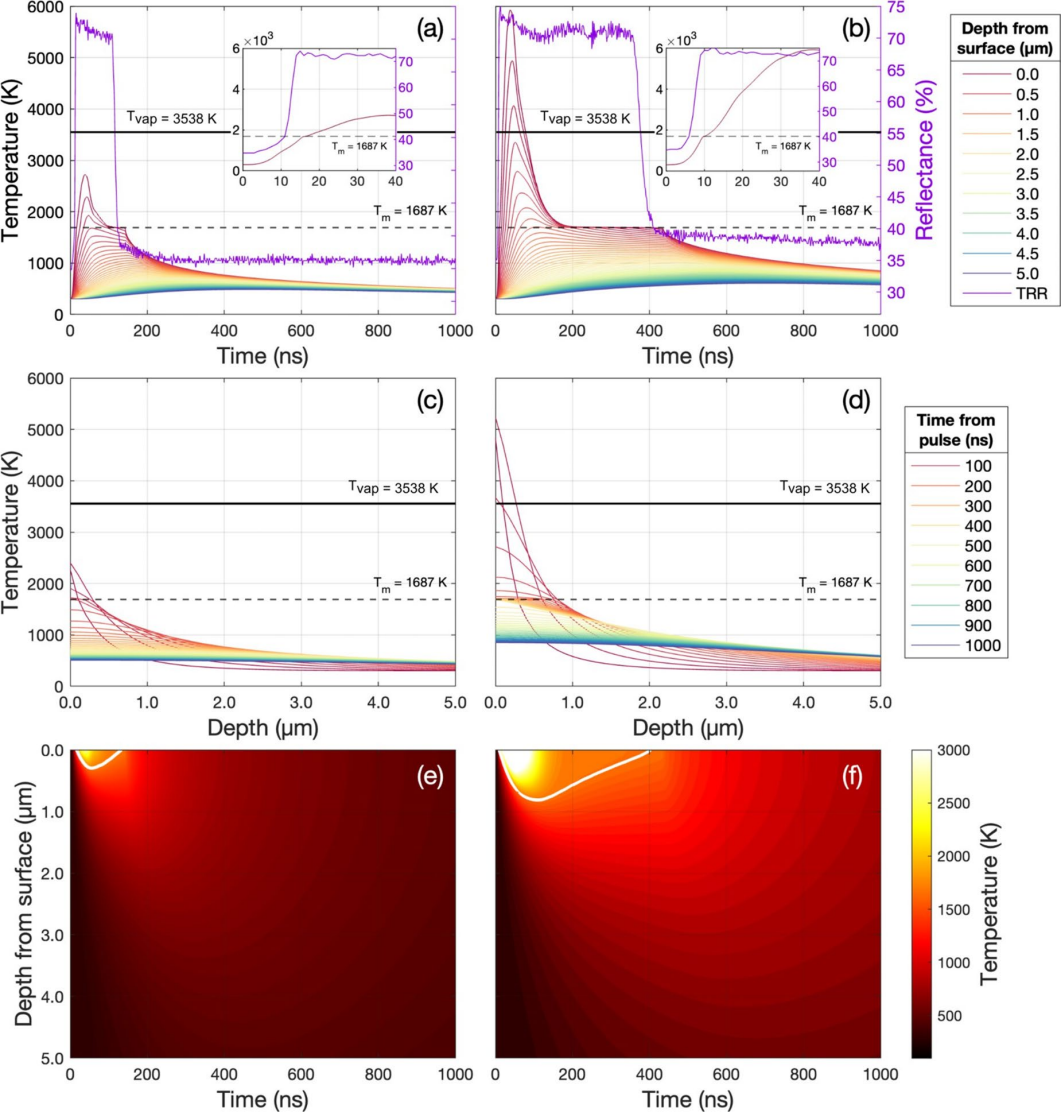
(**a**) Left axis: Optothermal simulations of the absolute temperature of virgin silicon as a function of time receiving a 1.5 J/cm^2^ excimer pulse. Simulations were performed at depth steps of 100 nm from 0 (surface) to 5 µm. Note, that the legend only shows every 500 nm. Right axis: Time-resolved reflectivity measurement of i-Si receiving a 1.5 J/cm^2^ excimer pulse. Inset: Initial 40 ns magnified to show simulated and measured solid–liquid phase transition. (**b**) Left axis: Optothermal simulations of the absolute temperature of virgin silicon as a function of time receiving a 2.8 J/cm^2^ excimer pulse. Right axis: Time-resolved reflectivity measurement receiving a 2.8 J/cm^2^ excimer pulse. Inset: Initial 40 ns magnified to show simulated and measured solid–liquid phase transition. (**c**) Optothermal simulations of the absolute temperature as a function of depth receiving a 1.5 J/cm^2^ excimer pulse. Simulations were performed in time steps of 0.1 ns from 0 to 1000 ns. Note, the plot shows data at 25 ns steps from 25 ns, and the legend only shows every 100 ns. (**d**) Optothermal simulations of the absolute temperature as a function of depth receiving a 2.8 J/cm^2^ excimer pulse. Simulations were performed in time steps of 0.1 ns from 0 to 1000 ns. Note, the plot displays data at 25 ns intervals from the first 25 ns, and the legend only shows every 100 ns. (**e**) Contour plot of the simulated absolute temperature as a function of time receiving a 1.5 J/cm^2^ excimer pulse with depth from the surface plotted on the reversed y-axis. An isoline line is included in white indicating the melt temperature of silicon (*T*_*m*_ = 1687 K), depicting the melt depth over time. (**f**) Contour plot of the simulated absolute temperature as a function of time receiving a 2.8 J/cm^2^ excimer pulse.

Extensive studies have been conducted on the interaction between Si and high-energy UV nanosecond pulsed lasers^37^. The rapid and intense heating of Si is achieved through light absorption. This process strongly depends on the dimensionless wavelength-dependent optical constants of the material; the refractive index, *n*_(*λ*=248 nm)_ = 1*.*6588 and extinction coefficient *k*_(*λ*=248 nm)_ = 3*.*617032^38^ (Fig. S11). *k* describes the loss of wave intensity to the material by defining the absorption coefficient *α* = 4*πk/λ*. For *λ* = 248 nm *α* = 1*.*83 × 10^6^ cm^*−*1^ (Fig. S12). The absorption edge of i-Si is determined by its band gap (*E*_*g*_) of 1.12 eV at 300 K, corresponding to *λ* = *hc/E*_*g*_ = 1107 nm. The photon energy at 248 nm (*E*_(248 nm)_ = 4.999 eV) exceeds the band gap of Si, hence the laser light that penetrates the material (refracted into the Si) is entirely absorbed (this corresponds to 32.7% of the initial laser light)^39^. This results in laser-induced electronic excitation leading to inter-band and intra-band transitions and photo-generated holes. The excited electrons contribute to multi-phonon cascade emission upon de-excitation, resulting in intense lattice vibrations and instantaneous local heating and initiating a complex sequence of processes including melting, ejection of atoms and ions, plasma initiation and expansion, and shock waves^40^. The plasma generated by a KrF excimer laser at the target surface is known to reach temperatures of 6000–10,000 K^41,42^. In HP-GIELD, the plasma is confined by the high-pressure gas environment, localising it at the sample surface and enhancing energy transfer and laser shock peening (LSP)^43^. The resultant effects are influenced by several factors, including pulse duration, energy, wavelength, the material properties of Si, and environmental conditions^44^. Beyond the 248 nm photon penetration depth (*δ*_*p*_ = 1*/α* = 5*.*46 nm), thermal diffusion becomes the dominant heat transfer mechanism in the material. Optothermal simulations were performed for a substrate of virgin i-Si receiving a single 248 nm excimer pulse at fluences of 1.5 and 2.8 J/cm^2^. The simulation outputs a matrix of the calculated temperature as a function of time for the initial 1000 ns in steps of 0.1 ns, and as a function of depth ranging from 0 (surface) to 5 µm in steps of 100 nm (Fig. 3a–d). We found that all light was effectively absorbed within the first 20 nm and thermal relaxation occurred after > 1 µs. The maximum surface temperature, *T*_*max*_, after one pulse of 1.5 and 2.8 J/cm^2^ were simulated to be 2720 and 5930 K, respectively, after *t *≈ 38 ns. However, since the opto-thermal simulation does not take into account the liquid to gas phase change, the temperatures calculated may be above the vaporisation temperature of Si (3538 K)^45^. In reality, it is not expected that the liquid will exceed the vaporization temperature of Si, hence the maximum temperature achieved by the liquid Si should be considered capped at 3538 K (indicated in Fig. 3a–d by the horizontal solid line at 3538 K). TRR measurements of i-Si receiving a 1.5 and 2.8 J/cm^2^ excimer pulse are included in Fig. 3a and b, respectively, for comparison to the optothermal simulation results. Insets of the initial 40 ns of the surface optothermal simulation and corresponding TRR measurement display the solid–liquid phase transition at the Si melt temperature (*T*_*m*_ = 1687 K), thus confirming the prediction of pulsed laser melting (PLM).

**Figure 4:**
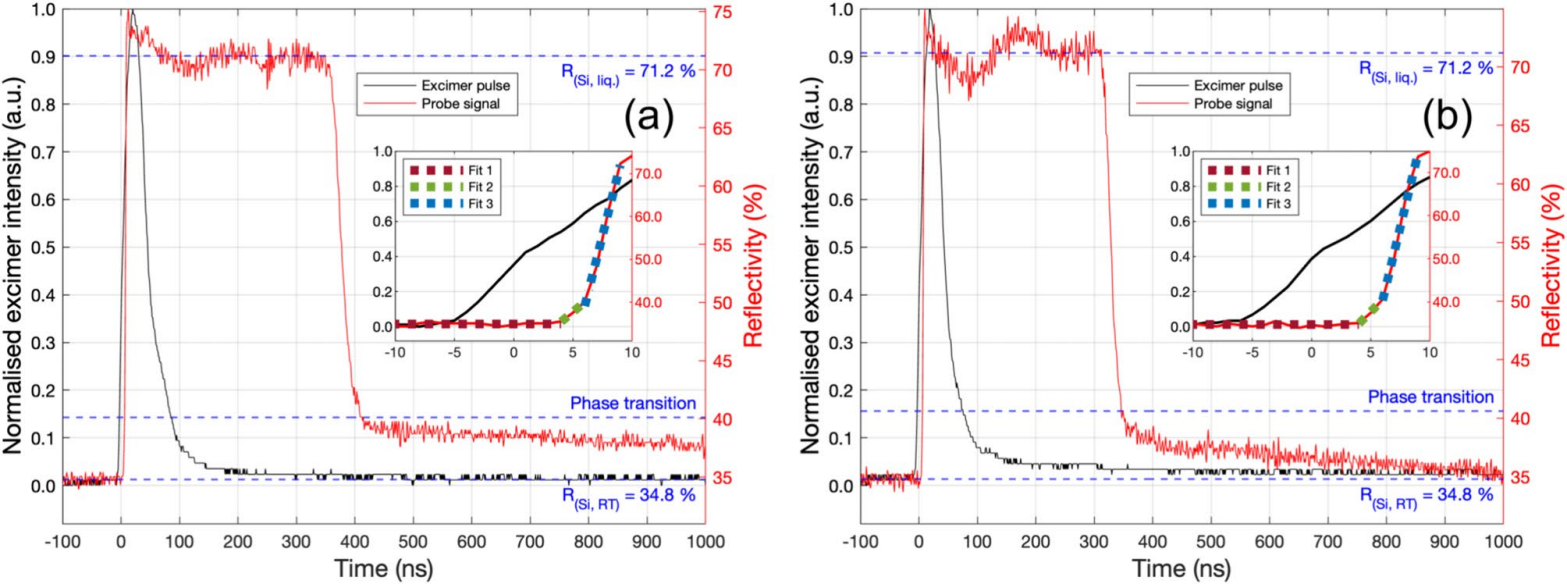
Excimer temporal intensity profile for a 2800 mJ/cm^2^ pulse (black) and time-resolved reflectivity of the Si surface at the centre of the irradiated area (red). **a** In atmosphere. **b** In 10 bar N_2_. The insets show linear regression fits (coloured dots) to three distinct regions of the reflectivity during the first 10 ns, relating to R_*Si, RT*_, a heating period, and solid–liquid phase transition.

TRR signature analysis of Si irradiated by one pulse of 2.8 J/cm^2^ in atmosphere and 10 bar N_2_ (Fig. 4a and b, respectively) was performed following the Lowndes and Wood model^46^. A 10 ± 1 ns delay was consistently observed between the 10 % rising edge of the normalised excimer intensity and *R*_*Si*_, displayed in the Fig. 4 inset. The reflectivity rise time, *τ*_*r*_, taken from the rising edge of the excimer pulse to the maximum reflectivity of liquid Si was measured to be 14 ± 1 ns, inclusive of the 10 ns rise delay. During the first 10 ns (insets of Fig. 4), three distinct regions were observed in *R*_*Si*_, each fit by linear regression (Table 2).

**Table 2:** Linear regression fit parameters with standard error and coefficient of determination of the Si phase transitions in air and 10 bar N_2_ measured by TRR in Fig. 4.

	Air			10 bar N_2_		
	Gradient (% ns^*−*1^)	Intercept (%)	R^2^	Gradient	Intercept	R^2^
Fit 1	0.0009 ± 0.0004	34.90 ± 0.01	0.9983	0.0015 ± 0.0005	34.76 ± 0.01	0.9971
Fit 2	2.31 ± 0.02	26.2 ± 0.1	0.9945	2.76 ± 0.03	23.9 ± 0.1	0.9932
Fit 3	10.96 ± 0.09	− 26.7 ± 0.6	0.9927	11.4 ± 0.1	-27.9 ± 0.7	0.9914

**Figure 5:**
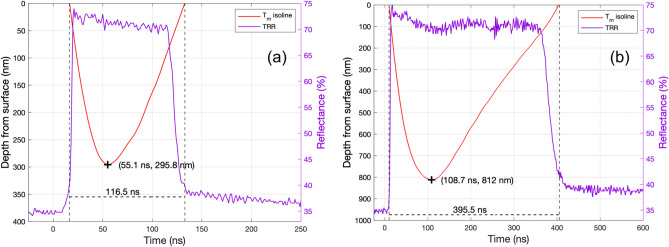
Left axes: Optothermal simulations isolated contour plot isoline at *T*_*m*_ = 1687 K for analysis of simulated peak melt depth and total melt duration. **a** 1.5 J/cm^2^ pulse, peak melt depth = 295.8 nm reached at 55.1 ns, total melt duration = 116.5 ns. **b** 2.8 J/cm^2^ pulse, peak melt depth = 812 nm reached at 108.7 ns, total melt duration = 395.5 ns. Right axes: TRR of the Si surface after a pulse of (**a**) 1.5 J/cm^2^ pulse and (**b**) 2.8 J/cm^2^.

Fit 1 (red dots) displays the initial constant average reflectivity of virgin Si at RT. Fit 2 (green dots) shows a positive slope as *R*_*Si*_ began to increase with a gradient of ≈ 2.5% ns^*−*1^ during a period of heating to the melt temperature, *T*_*m*_ = 1687 K, this occurred in ≈ 2–3 ns. Fit 3 (blue dots) has a second positive slope with a significantly steeper gradient of ≈ 11.2% ns^−1^ as the Si underwent the solid-liquid phase transition and *R*_*Si*_ rapidly climbed to *R*_*Si, liq.*_ within ≈ 3 ns. Phase change consistently took place at *R*_*Si*_ = 40*.*3 ± 0*.*3 %. In atmospheric conditions, *R*_*Si, liq.*_ was maintained for a duration, *τ*_*m*_, of 344 ± 2 ns, while in a 10 bar N_2_ environment *τ*_*m*_ was significantly shorter at 302 ± 2 ns. We discuss the onset of fixed-frequency oscillations in *R*_*Si,liq*._ of ≈ 6.67 MHz for fluences of 2.0 J/cm^2^ and above in the Supplementary Information (Figs. S16, S17). The fall time, *τ*_*f*_, measured as the time for *R*_*Si, liq.*_ fall back to the initial phase transition *R* was 53 ± 1 ns in atmosphere, and 37 ± 1 ns in 10 bar N_2_. The total melt duration, *τ* = *τ*_*m*_ + *τ*_*f*_, in atmosphere was 397 ± 2 ns, and 339 ± 2 ns in 10 bar N_2_. The characteristics of these TRR signatures thus confirm pulsed laser melting (PLM) in both atmosphere (as expected) and 10 bar N_2_. However, the high-pressure environment shortens the total melt duration by 58 ± 4 ns, or ≈ 15%. Contour plots of simulated temperature (colour bar) and depth from the surface (plotted on a reversed y-axis) as a function of time visualise the heat dispersion through the sample (Fig. 3e,f). An isoline line in white indicates *T*_*m*_, depicting the melt depth over time. The simulated peak melt depth and total melt duration are found by isolating the *T*_*m*_ isoline (Fig. 5). For a pulse of 1.5 J/cm^2^, a peak melt depth of 295.5 nm was reached after 55.1 ns with a total melt duration of 116.5 ns. For a pulse of 2.8 J/cm^2^, a peak melt depth of 812 nm was reached after 109 ns with a total melt duration of 395.5 ns. The corresponding TRR measurements included on the right axes show the simulated melt duration is in excellent agreement with those measured experimentally and confirms the simulation’s physicality.

We evaluated the Stokes–Einstein equation (Eq. 1)^47^ to determine the diffusion coefficient of N in Si at *T*_*max*_ = 5930 K. Where *k*_*B*_ is the Boltzmann constant, *η* is the temperature-dependent viscosity of Si derived through Sato et al.’s Arrhenius relation^48^ log(*η*) = − 0*.*727 + 819*/T*_*max*_ = 0*.*2577 mPa s, and *r* is the empirical atomic radius of N = 0.65 Å^49^. Thus, demonstrating these extreme temperatures allowed us to exceed N’s typical diffusivity in Si melt by over two orders of magnitude.

1 $$D(T_{max} ) = \frac{k_BT}{{6\pi \eta r}} = {2}.{59} \times {1}0^{{ - }{3}} {\text{cm}}^{{2}} {\text{s}}^{{ - }{1}}$$

**Figure 6:**
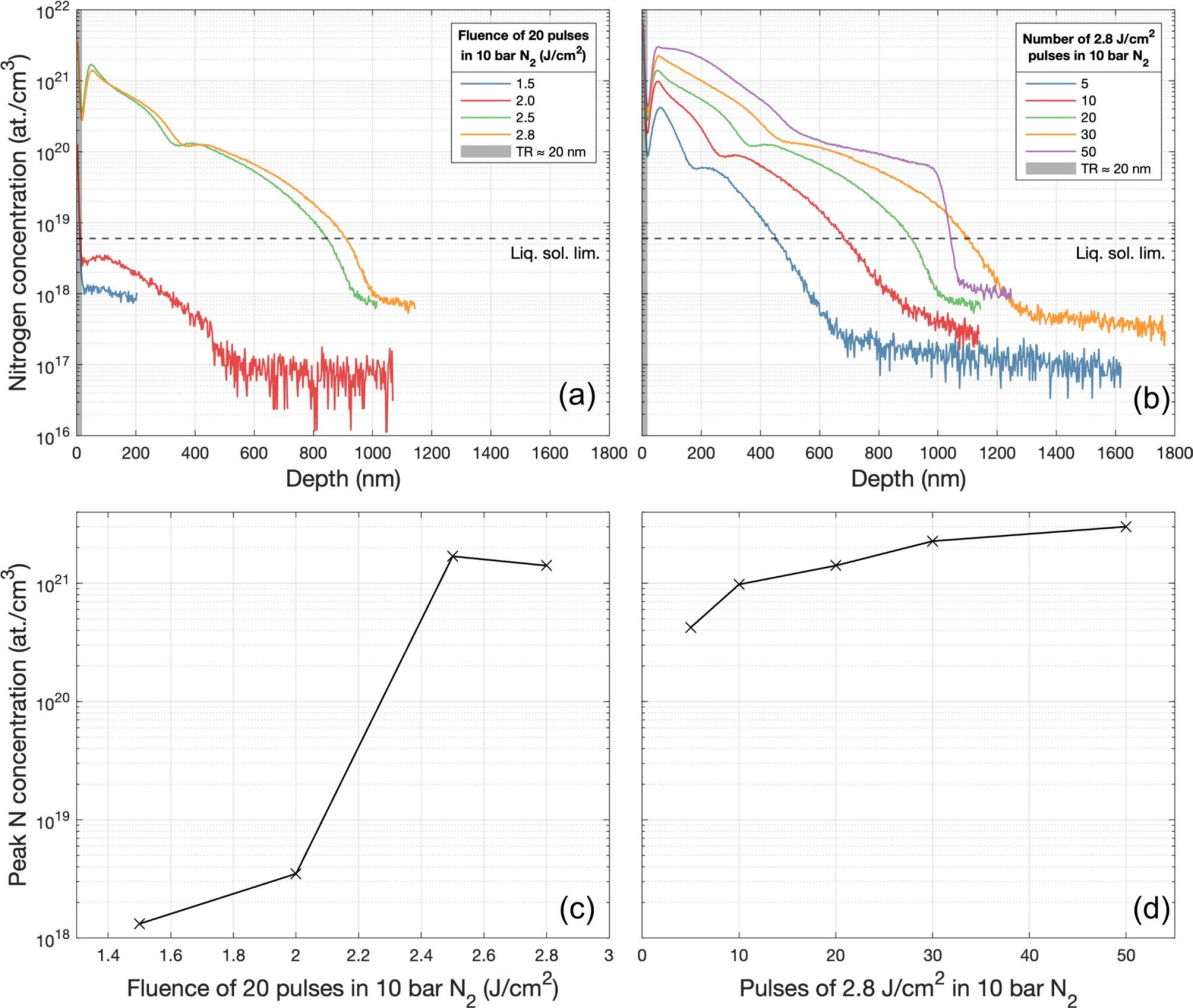
Top: ToF–SIMS nitrogen concentration depth profiles of intrinsic silicon processed with (**a**) 20 pulses of 1.5–2.8 J/cm^2^ in 10 bar N_2_. (**b**) 5–50 pulses of 2.8 J/cm^2^ in 10 bar N_2_. Bottom: Variation in peak nitrogen concentration as a function of (**c**) fluence of 20 pulses. (**d**) Number of pulses of 2.8 J/cm^2^.

The total concentration of N was investigated via depth profiling time-of-flight secondary ion mass spectrometry (Fig. 6a,b). We studied the effect of varying the number of pulses at a fixed fluence of 2.8 J/cm^2^ and the effect of varying the fluence at a fixed number of 20 pulses (all in 10 bar N2). A fluence threshold for significant N incorporation was identified, with over two orders of magnitude increase in concentration when the fluence is increased from 2.0 to 2.5 J/cm^2^. For 20 pulses of fluences ≥ 2.5 J/cm^2^, a peak N concentration of ≈ 1.6 × 10^21^ at cm^−3^ (3.2 at%) was observed at a depth of ≈ 50 nm (Fig. 6c). In all profiles for 20 pulses of fluences ≥ 2.5 J/cm^2^, a plateau in concentration consistently occurred after the initial peak between ≈ 350 and 450 nm before continuing to tail off as expected, the origin of this plateau, at present, is unknown. The N concentration in samples processed with 20 pulses of 2.5 J/cm^2^ was maintained above the liquid solubility limit to a depth of 850 nm, while that of samples processed with 2.8 J/cm^2^ was maintained 60 nm deeper to 910 nm. We achieved our highest measured total N concentration with 50 pulses of 2.8 J/cm^2^ in 10 bar N_2_ of 3.01 × 10^21^ at cm^−3^ (6.03 at%) at a depth ≈ 55 nm (Fig. 6d), ≈ 6 orders of magnitude greater than the solid solubility limit, confirming significant hyperdoping. For this sample, the maximum depth to which the N concentration is maintained above the liquid solubility limit was ≈ 1.1 µm, almost 300 nm beyond the simulated melt depth of 812 nm. T_*max*_ at 1.1 µm deep was simulated to reach 1411.35 K after ≈ 150 ns. This high temperature enabled the diffusion of N beyond the melt depth. Once 50 pulses are reached, the SIMS profile displayed a sharp decrease in concentration in the depth range 1.000–1.075 µm, the reason for which remains unclear. These results indicate that once the fluence threshold is overcome, it is the number of pulses that plays the largest role in determining the resultant nitrogen concentration depth profile. They also confirm that HP-GIELD achieves repeatable, very well-defined areas of hyperdoped nitrogen to micron-scale depths in a matter of nanoseconds. This is a significant result as, typically, to saturate liquid silicon to the solubility limit, one would have to expose it to N for many hours during growth uniformly. The origin of the fluence threshold may be elucidated through the melt depth and duration being on the order of 3 × greater for a 2.8 J/cm^2^ pulse, compared to a 1.5 J/cm^2^ pulse. Thus, despite achieving PLM with a 1.5 J/cm^2^ pulse, the lower diffusivity at *T*_*max*_ = 2720 K of *D*(*T*_*max*_) = 8*.*17 × 10^−4^ cm^2 ^s^−1^ combined with the significantly shorter period for which *T ≥ T*_*m*_ is maintained does not give sufficient opportunity for measurable concentrations of N to diffuse efficiently into the Si melt.

**Figure 7:**
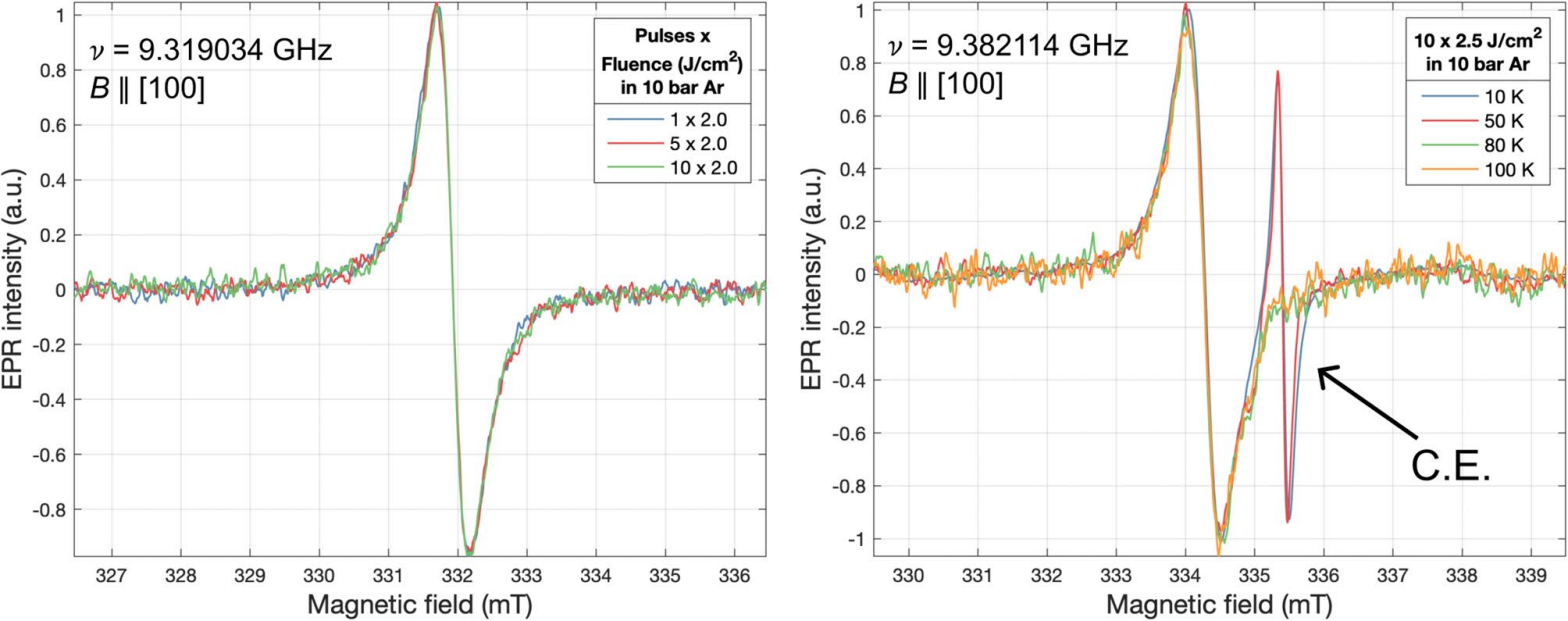
Left: RT EPR measurements of intrinsic silicon control samples processed in 10 bar Ar displaying only a single central line attributed to silicon dangling bonds. Right: Low-temperature EPR measurements of an intrinsic silicon control sample processed with 10 pulses of 2.5 J/cm^2^ in 10 bar Ar displaying the additional signal attributed to conduction electrons due to unidentified shallow donors.

EPR measurements of diced i-Si control samples processed in 10 bar Ar with 1, 5, and 10 pulses of 2.0 J/cm^2^ (Fig. 7 left) displayed a single line at RT which did not change in position or intensity while varying the number of pulses. Low-temperature (LT) measurements (Fig. 7 right) displayed an additional temperature-dependent isotropic signal right of centre, present for T ≤ 50 K.

**Figure 8:**
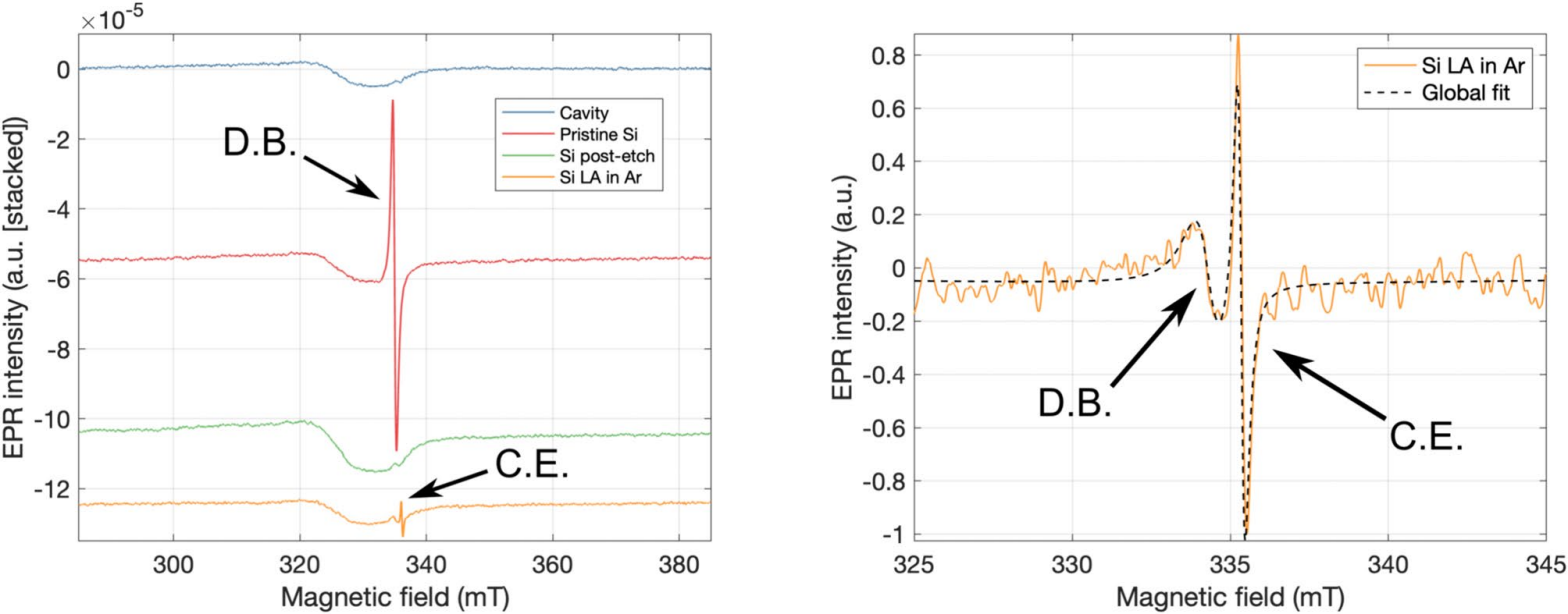
Left: Low-temperature (4.2 K) EPR measurements (stacked by Y offset) of the same intrinsic silicon control sample when pristine, after a 10 s etch in HF-HNO_3_ performed at RT, and after being laser annealed (LA) with 20 pulses of 2.8 J/cm^2^ in 10 bar Ar. While pristine, the signal due to silicon dangling bonds is present. Post-etch, the dangling bond signal is almost entirely removed. Post-laser processing in Ar, the additional signal attributed to conduction electrons due to unidentified shallow donors is introduced. N.B., the consistent dip observed in these spectra are a feature of the cavity used. Right: Zoomed plot of the LA in Ar spectrum after normalisation, baseline correction, and background subtraction. Included is the fit of both the C.E. and remaining D.B. EPR signals.

To uncover the source of the signal observed at RT, EPR measurements of a pristine diced i-Si sample were performed with *B* || [100] (Fig. 8). The consistent dip observed in these spectra was a feature of the cavity used (Fig. S18). These displayed the same single line at both RT and 4.2 K (Liquid helium, LHe) with *g* = 2.0055(1) and a peak-to-peak linewidth ∆*H*_*pp*_ = 0*.*55(5) mT, consistent with that of Si dangling bonds (D.B.). This signal could be removed by a RT isotropic wet etch for 10 s in a mixture of HF (48% assay)—HNO_3_ (65% assay) combined in a ratio of 5%/95% w/w, respectively, with an estimated etch rate of 11.5 µm per minute^50^. Thus, we attribute the *g* = 2*.*0055(1) line to damage introduced by dicing. To verify that our laser processing did not introduce any additional damage or dangling bonds, a HF-HNO_3_ etched control sample was processed in 10 bar Ar with 20 pulses of 2.8 J/cm^2^. At RT, no additional signals were introduced, therefore, supporting our claim that HP-GIELD did not create any dangling bonds. However, at 4.2 K, we again observed the addition of a line right of the D.B. signal with *g* = 1*.*9994(1) and ∆*H*_*pp*_ = 0*.*25(1) mT, which we attribute to conduction electrons (C.E.) caused by unidentified shallow donors.

**Figure 9:**
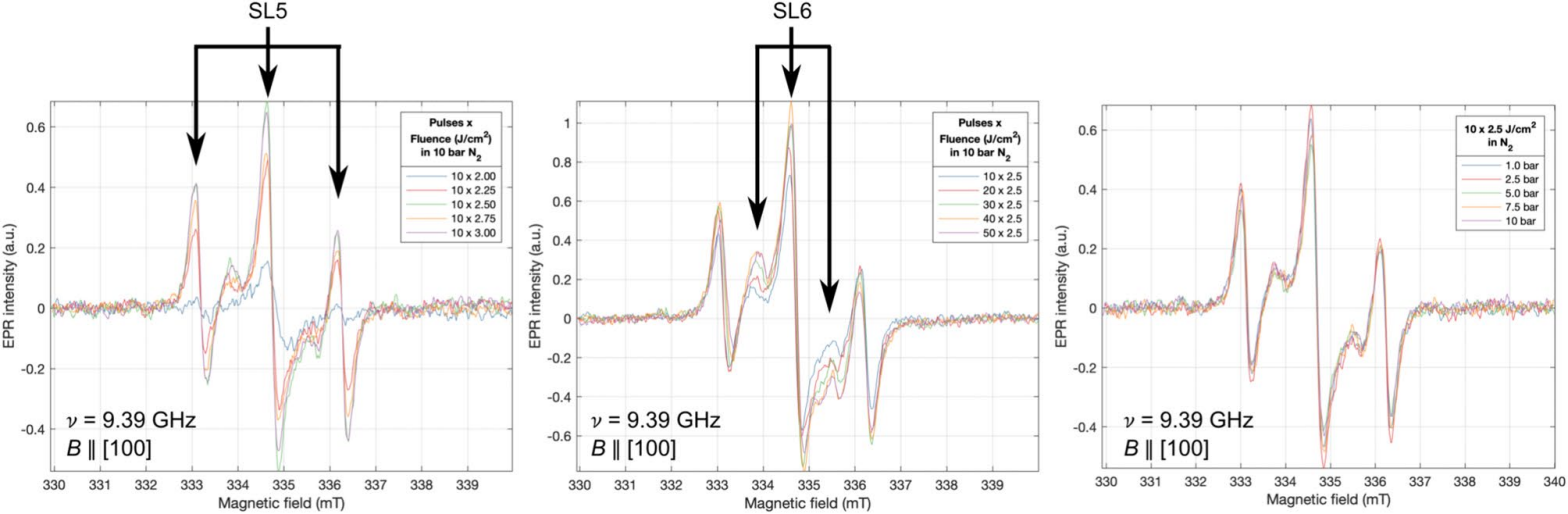
RT EPR measurements of Left: Intrinsic silicon samples processed in N_2_ with increasing fluence per pulse. Centre: increasing number of pulses per spot. Right: increasing N_2_ pressure.

The characteristic RT motionally-averaged N_*Si*_ signal, the so-called SL5 paramagnetic centre^24^ which consists of three resonances due to the nuclear spin *I* = 1 of ^14^N, was first observed in an i-Si sample processed with 10 pulses of 2.0 J/cm^2^ in 10 bar N_2_. Measurements performed at 77 K (Liquid nitrogen, LN_2_) reveal resonances consistent with the C_3*v*_ point symmetry of the centre. The additional line due to shallow donor conduction electrons was not seen in LT measurements of samples processed in N_2_, thus implying that N incorporation was compensating the shallow donors, a result consistent with electrical measurements. To optimise the fabrication process for the highest concentration of N_*Si*_ three sets of samples were processed, varying the fluence of 10 pulses in 10 bar N_2_, the number of pulses of 2.5 J/cm^2^ in 10 bar N_2_, and 10 pulses of 2.5 J/cm^2^ varying the N_2_ pressure (Fig. 9).

**Figure 10:**
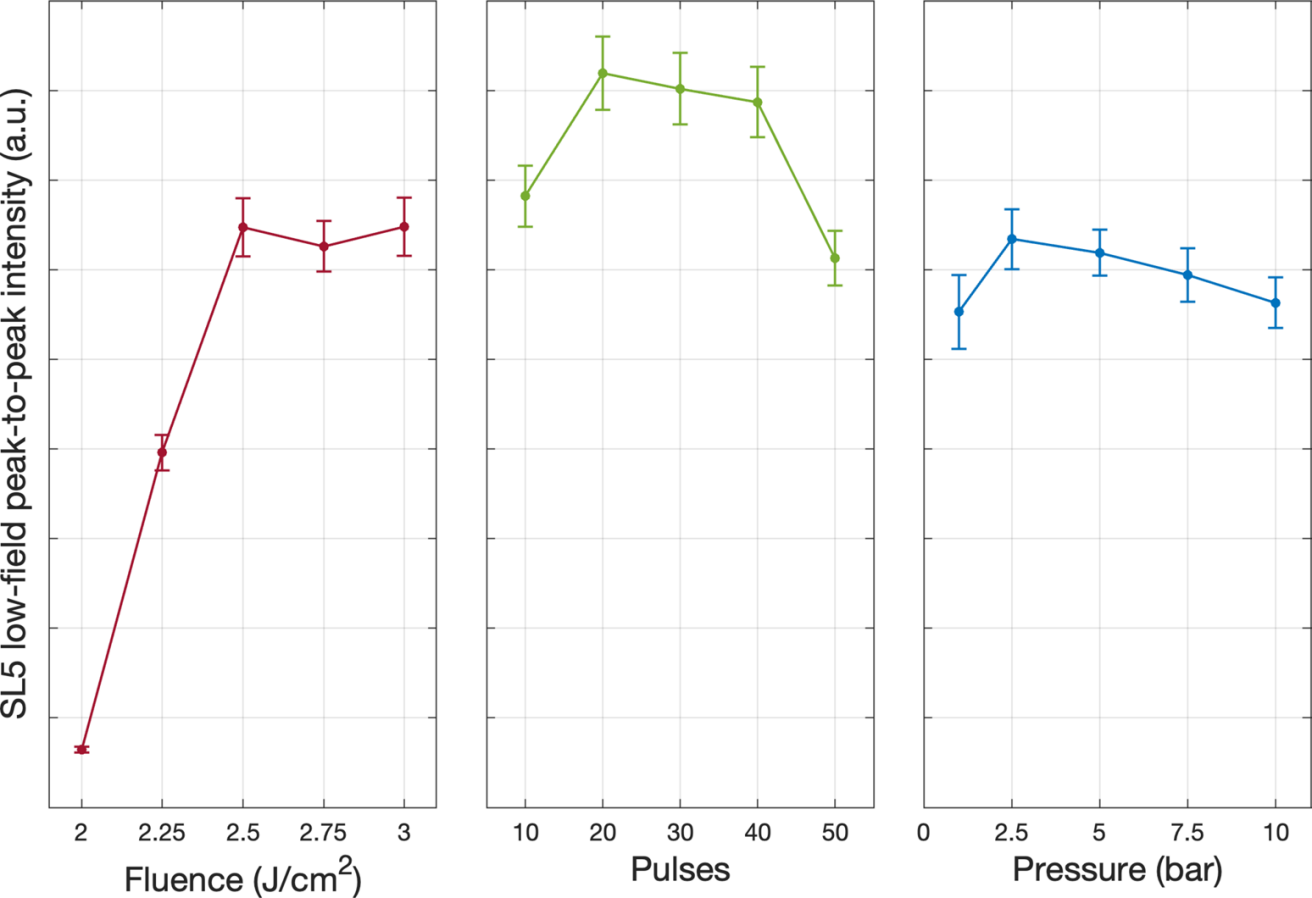
Quantitative peak-to-peak analysis of the SL5 low-field N_*Si*_ line of intrinsic silicon samples processed in N_2_ as Left: fluence, Centre: pulses, and Right: pressure were increased.

Three paramagnetic centres were detected in these samples. Firstly, the N_*Si*_ SL5 resonances, with the spin Hamiltonian parameters *g*_*||*_ = 2*.*0022(1)*, g* = 2*.*0083(2), ∆*H*_*pp*_ = 0*.*21(5) mT, and a hyperfine splitting *A*_*||*_ = 44*.*5(5)*, A* = 34*.*71(1) MHz. These parameters were determined through the fitting of measurements performed at 4.2 K in the SL5 freeze-out regime, rotating the sample in the cavity by 90°from [100] through [111] to [011] in increments of 5°and are in good agreement with Belli et al.^20^. This angular-dependence experiment also confirmed the signal’s C_3*v*_ point symmetry. In addition to the SL5 lines, a second set of three lines with a narrower hyperfine splitting emerged and grew in intensity with the number of pulses, consistent with the spin Hamiltonian parameters of the yet-assigned SL6 paramagnetic centre thought to be a nitrogen-related complex^24^. An intense central line suggests it comprises multiple absorptions. Microwave power saturation experiments confirmed the central line consisted of three components, the SL5, SL6, and dangling bond lines, which explained why it was consistently more intense. To analyse the effects of varying the processing parameters on the relative N_*Si*_ concentration, the low-field line peak-to-peak intensity was extracted as a function of processing parameters (Fig. 10).

**Figure 11:**
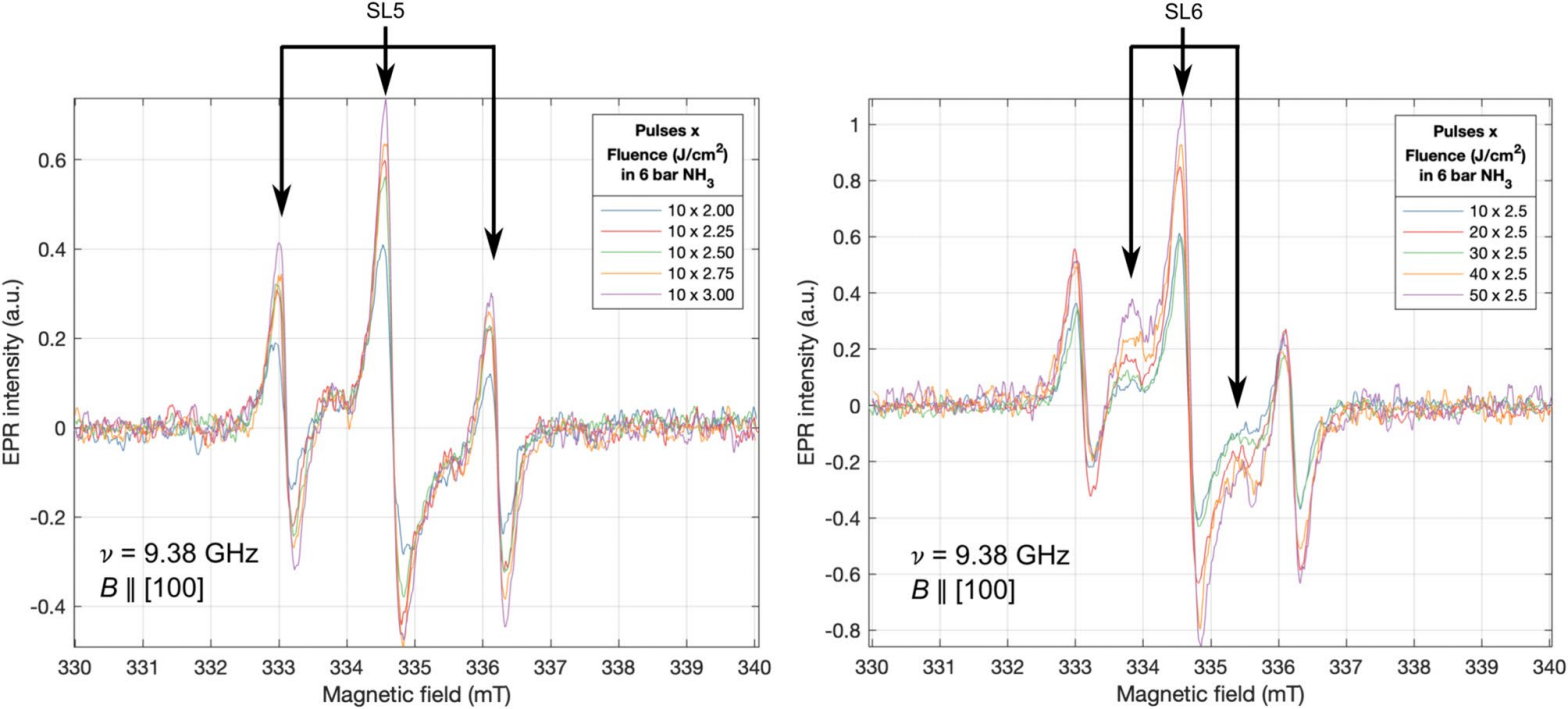
RT EPR measurements of intrinsic silicon samples processed in NH_3_ with Left: increasing fluence per pulse. Right: increasing the number of pulses per spot.

**Figure 12:**
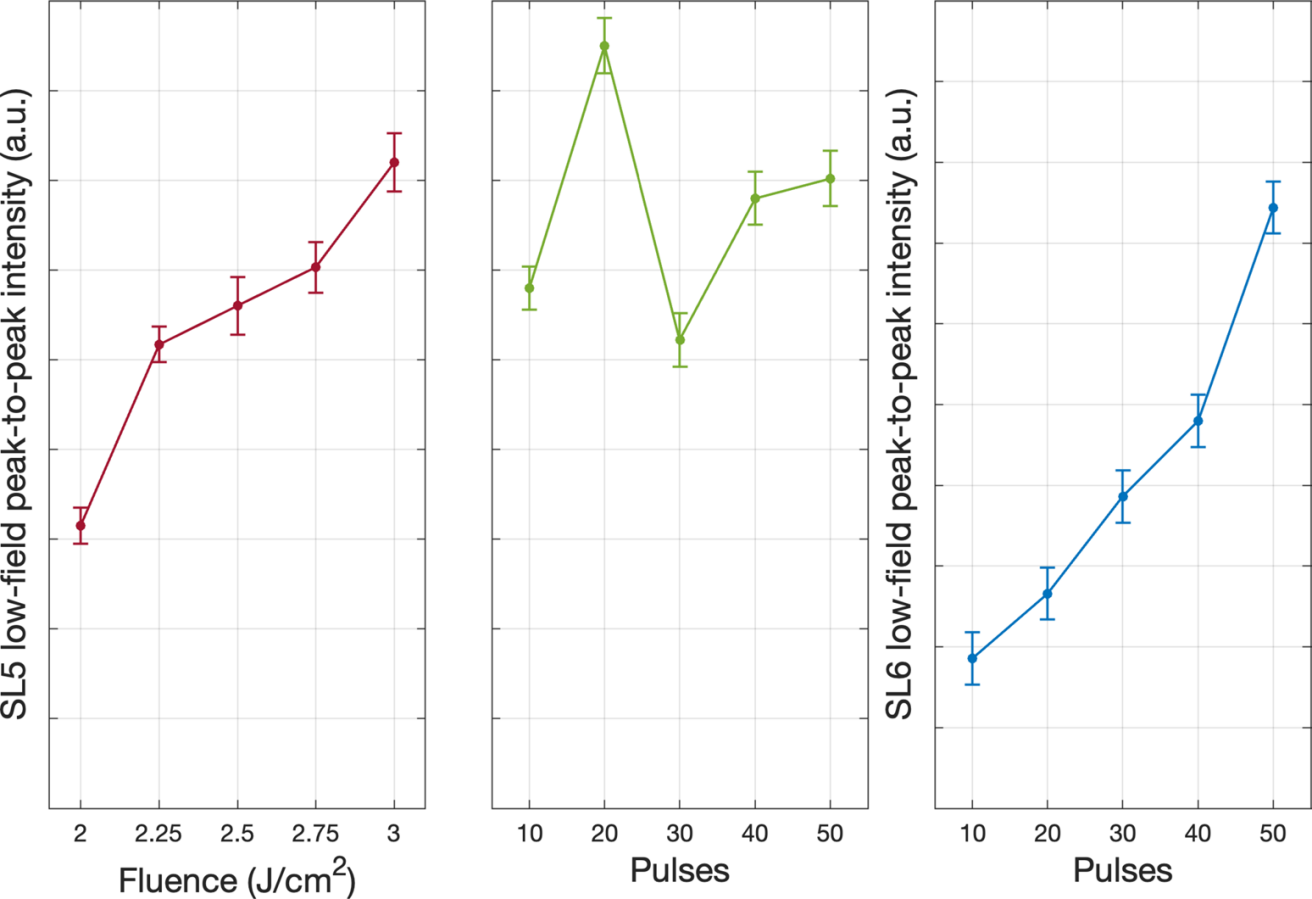
Quantitative peak-to-peak analysis of the: Left and Centre: SL5 low-field N_*Si*_ line of intrinsic silicon samples processed in NH_3_ as fluence and pulses are increased. Right: SL6 low-field N_*Si*_ line as pulses are increased.

We identified a fluence threshold of ≥2.5 J/cm^2^ to observe a significant N_*Si*_ signal, with the intensity increasing with fluence before plateauing at 2.5 J/cm^2^ and above, coherent with the SIMS results. The origin of this concentration plateau with increasing fluence above 2.5 J/cm^2^ may be due to a self-limiting shielding effect whereby large quantities of Si ions are ejected such that they shadow the surface from the laser beam. As the number of 2.5 J/cm^2^ pulses was increased from 10 to 50, the maximum low-field line peak-to-peak intensity occurred between 20 and 40 pulses. Changing the environment pressure did not appear to play any significant role in the N_*Si*_ signal intensity. This exercise was repeated in a more reactive environment of NH_3_ (Fig. 11). Varying the environmental pressure was omitted as high pressure is key in suppressing surface ablation, and it was identified that the pressure had no significant impact on the N_*Si*_ signal intensity in the N_2_ environment. These results display similar characteristics to those processed in N_2_, however, samples processed in NH_3_ achieve similar N_*Si*_ signal intensity at a lower fluence. The same three spin systems are again detected. Quantitative peak-to-peak analysis of the SL5 low-field line (Fig. 12 left and centre) and the SL6 low-field line intensity (Fig. 12 right) was performed on these spectra.

**Figure 13:**
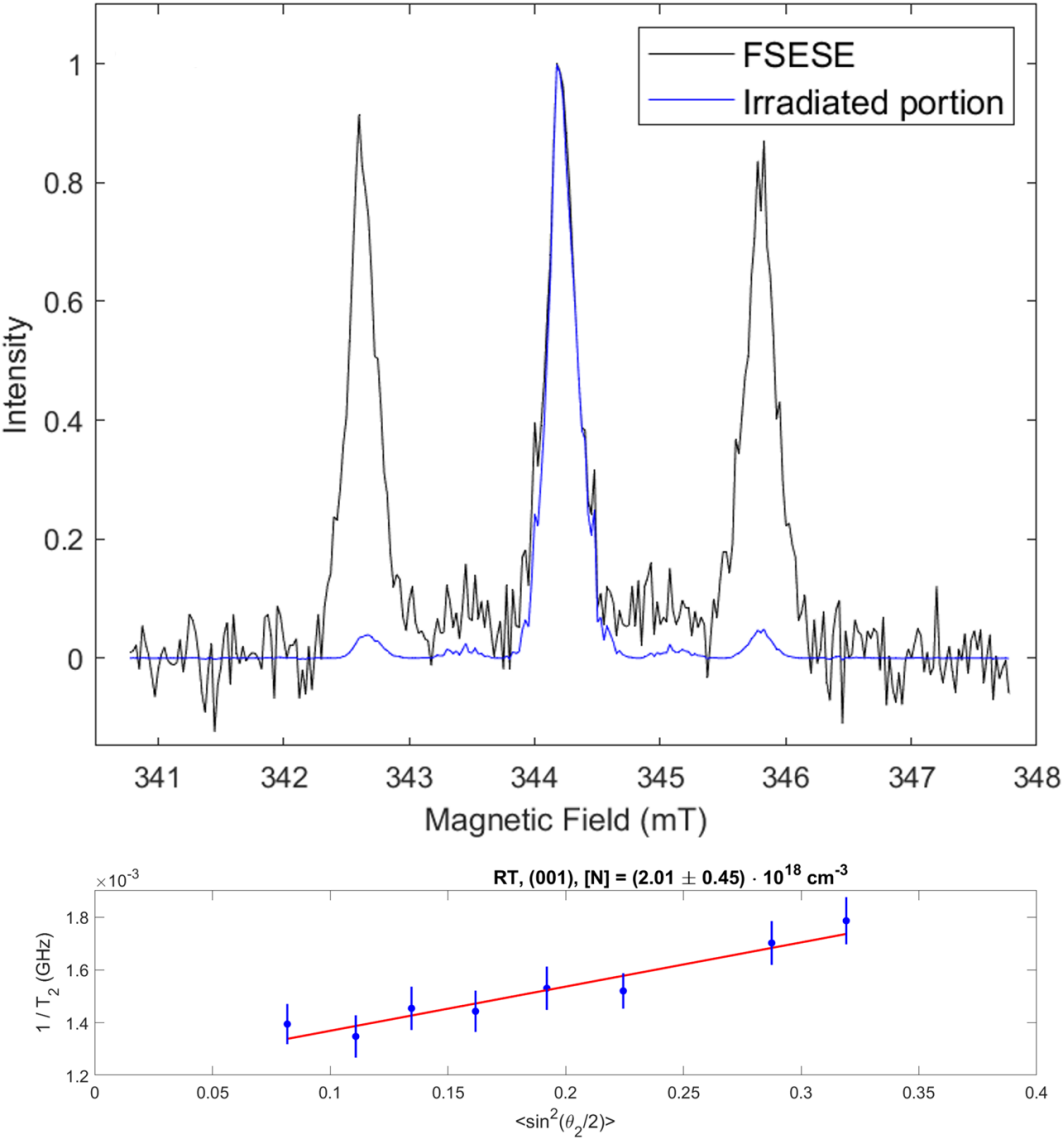
Pulsed EPR field-swept electron spin echo measurement of i-Si processed with 20 pulses of 2.8 J/cm^2^ in 10 bar N_2_ displaying a N_*Si*_ concentration of 2*.*0(5) × 10^18^at cm^*−*3^.

In both environments N_*Si*_ doping was successful. We identified several key thresholds and parameters that are optimal for maximising the SL5 signal intensity, and thus N_*Si*_ concentration. A fluence threshold, whereby increasing from 2.00 to 2.25 J/cm^2^ dramatically increased the N_*Si*_ signal. A damage threshold for fluences above 2.8 J/cm^2^, after which undulations and spiking of the silicon surface were generated (Figs. S19–S23). 20 pulses repeatably resulted in maximum SL5 signal intensity, slightly decreasing as pulses were further increased. However, SIMS measurements displayed a progressive and consistent increase with the number of pulses of 2.8 J/cm^2^ in total N concentration, the depth to which N was incorporated, and the depth at which the concentration plateau occurred after the initial peak. This trend follows the SL6 EPR signal, which increased sequentially with pulses (Fig. 12 right). We suggest that N_*Si*_ was first incorporated into the virgin lattice with fewer pulses, then, since N is known to interact strongly with vacancies in Si, as the pulses were increased beyond 20 it provided increased opportunity for the high concentration of N initially incorporated to diffuse toward vacancies and form N-V complexes, potentially explaining the increase in SL6 signal intensity as a function of pulses and providing further evidence to identify SL6 as an N-V complex defect. Therefore, we conclude that 20 pulses of 2.8 J/cm^2^ at the highest available environment pressure are the optimal fabrication parameters for maximising N_*Si*_ concentration. Pulsed EPR field-swept electron spin echo measurements of the SL5 signal allowed the determination of the specific concentration of substitutional nitrogen. For a sample of i-Si processed with 20 pulses of 2.8 J/cm^2^ in NH_3_ (Fig. 13) we achieved a N_*Si*_ concentration of 2*.*0(5) × 10^18^ at cm^*−*3^ (≈5*.*01 ×10^*−*3^ at%), thus confirming HP-GIELD achieves hyperdoping of not only total N concentration, but specifically hyperdoping of N_*Si*_. Comparing this result with the SIMS N concentration (1*.*41 × 10^21^ at cm^*−*3^ or 2.823 at%) of a sample fabricated under similar conditions enables an estimation of the activation efficiency of N_*Si*_, as well as the concentration of interstitial N. In this instance, the interstitial N concentration is ≈1*.*4075 × 10^21^ at cm^*−*3^ (or 2.818 at%) and the maximum activation efficiency was found to be only 0.18%, highlighting the persistent challenges that beset attempts to hyperdope Si with N.

The SL6 signal will be discussed further in a forthcoming paper which aims to analyse the defect symmetry and freeze-out temperature in more detail, along with performing complementary electrical characterisation of both SL5 and SL6, and infrared spectroscopic ellipsometry (IRSE) measurements to identify the known IR local vibrational modes (LVM) of nitrogen defects in silicon.

## Conclusions

We demonstrated the successful fabrication of nitrogen-hyperdoped silicon using our high-pressure gas immersion excimer laser doping (HP-GIELD) technique in nitrogen-rich environments. By optimising the processing parameters, samples fabricated using HP-GIELD exhibited total nitrogen concentrations surpassing 6 at% (3.01 × 10^21^ at/cm^3^), 12 times greater than the highest previously reported, and six orders of magnitude beyond the solid-solubility limit. Additionally, we achieved hyperdoping of the illusive N_*Si*_ defect to a concentration of 2*.*0(5) × 10^18 ^at cm^−3^ (4.1 × 10^*−*3^ at%), three orders of magnitude greater than the solid-solubility limit. Our method involved only a single processing step, eliminating the need for common pre-fabrication treatments such as HF native oxide removal, ion implantation, and thermal annealing. An NH_3_ environment yielded the highest overall N_*Si*_ EPR signal intensity with 20 pulses of 2.8 J/cm^2^ and accomplished the same signal intensity at lower fluences compared to a N_2_ environment, thereby minimising potential laser-induced surface damage. We propose that hydrogen provided by NH_3_ undergoes a redox reaction with native silicon oxide, resulting in the removal of oxygen. Nitrogen is also known to enhance the precipitation of oxygen in silicon. Both of these effects may contribute to greater nitrogen diffusion into the lattice. We identified a minimum fluence threshold of 2.5 J/cm^2^ which must be reached for significant nitrogen doping, and elucidated its origin to be due to the temperature-dependent diffusivity and melt duration which are strongly contingent on fluence. We also identified a nitrogen concentration plateau when increasing the fluence beyond 2.5 J/cm^2^. This may be due to a shielding effect of the ionised mass of vaporised Si, i.e., above the identified fluence damage threshold of 3.0 J/cm^2^, large quantities of silicon ions are ejected such that they shadow the Si surface from the laser beam and self-limit the process. We demonstrated the controllable fabrication of a nitrogen-related EPR triplet with narrower hyperfine splitting than the substitutional nitrogen SL5 signal, consistent with the Hamiltonian spin parameters of the yet-assigned SL6 paramagnetic centre which is thought to be a nitrogen-vacancy complex. SIMS measurements of samples processed with an increasing number of pulses provided further evidence to identify SL6 as a N–V complex. We suggest that N_*Si*_ is first incorporated into the virgin lattice with fewer pulses, then, through the known strong interaction between the N_*Si*_ defects and vacancies in the silicon lattice, as the pulses were increased beyond 20 it provided increased opportunity for the high concentration of nitrogen initially incorporated to diffuse toward vacancies and form N–V complexes. We presented an optothermal model which described the laser-matter interaction encompassing photon absorption, temperature-dependent thermal diffusion, and phase changes, and investigated subsequent atomic diffusion of nitrogen in silicon through a comparative analysis of simulation results and experimentally measured melt duration and composition. Based on current data we propose a doping mechanism whereby pulsed laser melting and ejection of atoms and ions result in the ignition and expansion of an intense plasma which is confined by the high-pressure environment. This highly energetic process liberates nitrogen molecules from the local gas environment. Subsequently, thermal expansion stresses, laser shock peening, and vibrational excitations localised at the substrate surface facilitate enhanced nitrogen diffusion through the liquid silicon and greatly amplify diffusion processes to achieve hyperdoping without the generation of Si dangling bonds.

### Supplementary Information

Supplementary Information.

## Data Availability

All data that support the findings of this study are included in the article (and the supplementary information).

## References

[1] Tong, Z., Bu, M., Zhang, Y., Yang, D. & Pi, X. Hyperdoped silicon: Processing, properties, and devices. *J. Semicond.* **43**, 093101. 10.1088/1674-4926/43/9/093101 (2022). 10.1088/1674-4926/43/9/093101

[2] Abe, T., Kikuchi, K., Shirai, S. & Murakoa, S. Impurities in silicon single crystals—A current view. In *Semiconductor Silicon 1981: Proceedings of the Fourth International Symposium on Silicon Materials Science and Technology* Vol. 815 (eds Huff, H. R. *et al.*) 54–71 (Springer, 1981).

[3] Stein, H. J. Vibrational absorption bands for implanted nitrogen in crystalline silicon. *Appl. Phys. Lett.* **43**, 296–298. 10.1063/1.94291 (1983). 10.1063/1.94291

[4] Stein, H. J. Infrared absorption band for substitutional nitrogen in silicon. *Appl. Phys. Lett.* **47**, 1339–1341. 10.1063/1.96273 (1985). 10.1063/1.96273

[5] Jones, R., Öberg, S., Berg Rasmussen, F. & Bech Nielsen, B. Identification of the dominant nitrogen defect in silicon. *Phys. Rev. Lett.* **72**, 1882–1885. 10.1103/PhysRevLett.72.1882 (1994). 10055728 10.1103/PhysRevLett.72.1882

[6] Goss, J. P., Hahn, I., Jones, R., Briddon, P. R. & Öberg, S. Vibrational modes and electronic properties of nitrogen defects in silicon. *Phys. Rev. B Condens. Matter. Mater. Phys.*10.1103/PhysRevB.67.045206 (2003). 10.1103/PhysRevB.67.045206

[7] Sgourou, E. N., Angeletos, T., Chroneos, A. & Londos, C. A. Infrared study of defects in nitrogen-doped electron irradiated silicon. *J. Mater. Sci. Mater. Electron.* **27**, 2054–2061. 10.1007/s10854-015-3991-2 (2016). 10.1007/s10854-015-3991-2

[8] Platonenko, A. *et al.* Nitrogen interstitial defects in silicon. A quantum mechanical investigation of the structural, electronic and vibrational properties. *Mater. Today Commun.*10.1016/j.mtcomm.2019.100616 (2019). 10.1016/j.mtcomm.2019.100616 31524895

[9] Platonenko, A. *et al.* Nitrogen substitutional defects in silicon. A quantum mechanical investigation of the structural, electronic and vibrational properties. *Phys. Chem. Chem. Phys.* **21**, 20939–20950. 10.1039/C9CP03185E (2019). 31524895 10.1039/C9CP03185E

[10] Wang, W. *et al.* NO2 gas sensor with excellent performance based on thermally modified nitrogen-hyperdoped silicon. *Sensors Actuators B: Chem.* **35**, 4. 10.1016/j.snb.2021.131193 (2022). 10.1016/j.snb.2021.131193

[11] Potsidi, M. S. *et al.* Theoretical investigation of nitrogen-vacancy defects in silicon. *AIP Adv.*10.1063/5.0075799 (2022). 10.1063/5.0075799

[12] Sumino, K., Yonenaga, I., Imai, M. & Abe, T. Effects of nitrogen on dislocation behavior and mechanical strength in silicon crystals. *J. Appl. Phys.* **54**, 5016–5020. 10.1063/1.332770 (1983). 10.1063/1.332770

[13] Ma, X., Yu, X., Fan, R. & Yang, D. Formation of pnp bipolar structure by thermal donors in nitrogen-containing p-type Czochralski silicon wafers. *Appl. Phys. Lett.* **81**, 496–498. 10.1063/1.1494466 (2002). 10.1063/1.1494466

[14] Voronkov, V. V. *et al.* Shallow thermal donors in nitrogen-doped silicon. *J. Appl. Phys.* **89**, 4289–4293. 10.1063/1.1356436 (2001). 10.1063/1.1356436

[15] Yang, D., Chu, J., Xu, J. & Que, D. Behavior of oxidation-induced stacking faults in annealed Czochraiski silicon doped by nitrogen. *J. Appl. Phys.* **93**, 8926–8929. 10.1063/1.1569978 (2003). 10.1063/1.1569978

[16] Nakai, K. *et al.* Oxygen precipitation in nitrogen-doped Czochralski-grown silicon crystals. *J. Appl. Phys.* **89**, 4301–4309. 10.1063/1.1356425 (2001). 10.1063/1.1356425

[17] Zhu, Z. *et al.* Electronic band structure and sub-band-gap absorption of nitrogen hyperdoped silicon. *Sci. Rep.*10.1038/srep10513 (2015). 26012369 10.1038/srep10513 PMC4444955

[18] Tokumaru, Y., Okushi, H., Masui, T. & Abe, T. Deep levels associated with nitrogen in silicon. *Jpn. J. Appl. Phys.* **21**, L443. 10.1143/JJAP.21.L443 (1982). 10.1143/JJAP.21.L443

[19] Sze, S. & Ng, K. K. *Physics of Semiconductor Devices* 3rd edn. (Wiley, 2006).

[20] Belli, M., Fanciulli, M. & Batani, D. Electron spin resonance of substitutional nitrogen in silicon. *Phys. Rev. B Condens. Matter Mater. Phys.*10.1103/PhysRevB.89.115207 (2014). 10.1103/PhysRevB.89.115207

[21] Kane, B. E. A silicon-based nuclear spin quantum computer. *Nature* **393**, 133–137. 10.1038/30156 (1998). 10.1038/30156

[22] Yadav, P., Arora, H. & Samanta, A. Nitrogen in silicon for room temperature single-electron tunneling devices. *Appl. Phys. Lett.* **122**, 083502. 10.1063/5.0136182 (2023). 10.1063/5.0136182

[23] Milnes, A. G. Semiconductor physics: Deep impurities in semiconductors. *Science* **183**, 1–526. 10.1126/science.183.4131.1284.a (1973). 10.1126/science.183.4131.1284.a

[24] Brower, K. L. Deep-level nitrogen centers in laser-annealed ion-implanted silicon. *Phys. Rev. B* **26**, 6040–6052. 10.1103/PhysRevB.26.6040 (1982). 10.1103/PhysRevB.26.6040

[25] Murakami, K., Masuda, K., Aoyagi, Y. & Namba, S. Experimental tests of non-thermal effect for pulsed-laser annealing by time-resolved reflectivity and EPR measurements. *Physica B+C.* **116**, 564–569. 10.1016/0378-4363(83)90308-X (1983). 10.1016/0378-4363(83)90308-X

[26] Yatsurugi, Y., Akiyama, N., Endo, Y. & Nozaki, T. Concentration, solubility, and equilibrium distribution coefficient of nitrogen and oxygen in semiconductor silicon. *J. Electrochem. Soc.* **120**, 975. 10.1149/1.2403610 (1973). 10.1149/1.2403610

[27] Luo, J., Zhou, C., Li, Q., Cheng, Y. & Liu, L. Diffusion coefficients of carbon, oxygen and nitrogen in silicon melt. *J. Cryst. Growth*10.1016/j.jcrysgro.2021.126476 (2022). 10.1016/j.jcrysgro.2021.126476

[28] Mitchell, J. B., Shewchun, J., Thompson, D. A. & Davies, J. A. Nitrogen-implanted silicon. II. Electrical properties. *J. Appl. Phys.* **46**, 335–343. 10.1063/1.321340 (1975). 10.1063/1.321340

[29] Murakami, K., Itoh, H., Takita, K. & Masuda, K. Substitutional nitrogen impurities in pulsed-laser annealed silicon. *Appl. Phys. Lett.* **45**, 176–178. 10.1063/1.95160 (1984). 10.1063/1.95160

[30] Jones, R., Hahn, I., Goss, J. P., Briddon, P. R. & Öberg, S. Structure and electronic properties of nitrogen defects in silicon. *Solid State Phenom.* **95–96**, 93–98. 10.4028/www.scientific.net/SSP.95-96.93 (2003). 10.4028/www.scientific.net/SSP.95-96.93

[31] Brower, K. L. Jahn-Teller-distorted nitrogen donor in laser-annealed silicon. *Phys. Rev. Lett.* **44**, 1627–1629. 10.1103/PhysRevLett.44.1627 (1980). 10.1103/PhysRevLett.44.1627

[32] Dong, X. *et al.* A nitrogen-hyperdoped silicon material formed by femtosecond laser irradiation. *Appl. Phys. Lett.* **104**, 091907. 10.1063/1.4868017 (2014). 10.1063/1.4868017

[33] Greenwood, N. N. & Earnshaw, A. *Chemistry of the Elements* 2nd edn. (Butterworth-Heinemann, 1997).

[34] Zhou, A. F. UV excimer laser beam homogenization for micromachining applications. *Opt. Photon. Lett.* **04**, 75–81. 10.1142/S1793528811000226 (2011). 10.1142/S1793528811000226

[35] Diez, M., Ametowobla, M. & Graf, T. Time-resolved reflectivity and temperature measurements during laser irradiation of crystalline silicon. *J. Laser Micro/Nanoeng.* **12**, 230–234. 10.2961/jlmn.2017.03.0010 (2017). 10.2961/jlmn.2017.03.0010

[36] Stoll, S. & Schweiger, A. EasySpin, a comprehensive software package for spectral simulation and analysis in EPR. *J. Magn. Reson.* **178**, 42–55. 10.1016/j.jmr.2005.08.013 (2006). 16188474 10.1016/j.jmr.2005.08.013

[37] Yoo, J. H., Jeong, S. H., Greif, R. & Russo, R. E. Explosive change in crater properties during high power nanosecond laser ablation of silicon. *J. Appl. Phys.* **88**, 1638–1649. 10.1063/1.373865 (2000). 10.1063/1.373865

[38] Herzinger, C. M., Johs, B., McGahan, W. A., Woollam, J. A. & Paulson, W. Ellipsometric determination of optical constants for silicon and thermally grown silicon dioxide via a multi-sample, multi-wavelength, multi-angle investigation. *J. Appl. Phys.* **83**, 3323–3336. 10.1063/1.367101 (1998). 10.1063/1.367101

[39] Kasap, S. O. *Electronic Materials & Devices* 4th edn. (McGraw Hill, 2017).

[40] Zeng, X. J., Mao, X., Greif, R. & Russo, R. E. Ultraviolet femtosecond and nanosecond laser ablation of silicon: ablation efficiency and laser-induced plasma expansion. In Phipps, C. R. (ed.) *High-Power Laser Ablation V*, *Society of Photo-Optical Instrumentation Engineers (SPIE) Conference Series*, vol. 5448, 1150–1158. 10.1117/12.544401 (2004).

[41] Lu, Y.-F., Tao, Z.-B. & Hong, M.-H. Characteristics of excimer laser induced plasma from an aluminum target by spectroscopic study. *Jpn. J. Appl. Phys.* **38**, 2958. 10.1143/JJAP.38.2958 (1999). 10.1143/JJAP.38.2958

[42] He, X., Hu, W., Li, C., Guo, L. & Lu, Y. Generation of high-temperature and low-density plasmas for improved spectral resolutions in laser-induced breakdown spectroscopy. *Opt. Express* **19**, 10997. 10.1364/OE.19.010997 (2011). 21643361 10.1364/OE.19.010997

[43] Fabbro, R., Fournier, J., Ballard, P., Devaux, D. & Virmont, J. Physical study of laser-produced plasma in confined geometry. *J. Appl. Phys.* **68**, 775–784. 10.1063/1.346783 (1990). 10.1063/1.346783

[44] Russo, R. E., Mao, X. & Mao, S. S. The physics of laser ablation in microchemical analysis. *Anal. Chem.* **74**, 70A-77A. 10.1021/ac0219445 (2002). 11838700 10.1021/ac0219445

[45] National Center for Biotechnology Information. PubChem Element Summary for AtomicNumber 14, Silicon (2024).

[46] Lowndes, D. H. & Wood, R. F. Time-resolved reflectivity during pulsed-laser irradiation of GaAs. *Appl. Phys. Lett.* **38**, 971–973. 10.1063/1.92239 (1981). 10.1063/1.92239

[47] Einstein, A. On the movement of small particles suspended in a stationary liquid demanded by the molecular-kinetic theory of heat. *Annalen der Physik* **322**, 549–560. 10.1002/andp.19053220806 (1905). 10.1002/andp.19053220806

[48] Sato, Y. *et al.* Viscosity of molten silicon and the factors affecting measurement. *J. Cryst. Growth* **249**, 404–415. 10.1016/S0022-0248(02)02153-X (2003). 10.1016/S0022-0248(02)02153-X

[49] Slater, J. C. Atomic radii in crystals. *The J. Chem. Phys.* **41**, 3199–3204. 10.1063/1.1725697 (1964). 10.1063/1.1725697

[50] Zhang, X. G. Electrochemistry of Silicon Etching. In *Encyclopedia of Electrochemistry*, 10.1002/9783527610426. bard050011 (Wiley, 2007).

