# Supplementary material for: Fabrication of nitrogen-hyperdoped silicon by high-pressure gas immersion excimer laser doping
Source: Sci Rep. 2024 Aug 23;14:19640. doi: 10.1038/s41598-024-69552-8 (PMC11344057; doi:10.1038/s41598-024-69552-8)
Supplement: Supplementary file 1 — Supplementary Information. [file 41598_2024_69552_MOESM1_ESM.pdf]

Supplementary information

Laser dicing

An optimal number of 300 passes at 100 % power (12.7 kW peak pulse power), fired at 20 kHz, with a beam deflection velocity of 100 mm/s resulting in an 80 % overlap of the 25 µm diameter spots [Figure S1] was chosen to dice samples for this study, which cut ≈ 90 % through the wafer from the backside [Figure S2] and kept the individual die attached to one another during the process yet allowed for easy separation and maximum yield [Figure S3]. The front side of the wafer was affixed to dicing tape to protect the surface of interest during dicing. The die were released from the dicing tape and had dust and debris removed via a RT solvent cleaning procedure, whereby die were agitated by an ultrasonic cleaner for five minutes while immersed in acetone, followed by methanol, and then blow-dried with nitrogen.

The 532 nm Trumpf Trumark laser is pulsed and has a measured pulse duration of ≈ 20 ns, a peak pulse power of 12.7 kW, and is fired at 20 kHz (F). The focused laser spot is circular and ≈ 25 µm in diameter (d). A beam deflection velocity of 100 mm/s (v) was used, resulting in an 80 % spot overlap (o) [Equation S1, Figure S1].

$$o\ (\%) = \left(1 - \frac{v}{d \times F}\right) \times 100 \tag{S1}$$

$$= \left(1 - \frac{100\ \text{mm/s}}{25\ \mu\text{m} \times 20\ \text{kHz}}\right) \times 100 = 80\ \% \tag{S2}$$

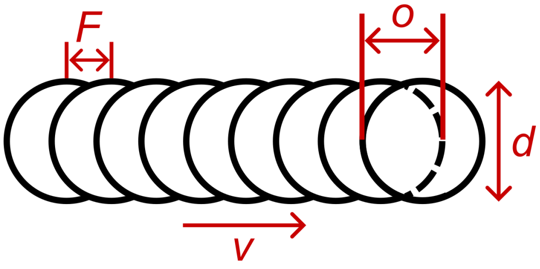

Figure S1. Schematic visualising the dicing laser pulse overlap.

Die scribed with 1 - 300 passes did not separate during marking. 100 - 300 passes were easily separated with tweezers, while 400+ passes cut completely through the wafer. Images taken of the edge of the die using a Lumenera Infinity2 microscope camera demonstrate a linearly increasing depth of cut with increasing passes through a reduction in the cleaved surface (bright areas) in Figure S2. The scribe depth was measured using Fiji (ImageJ), and calibrated against the known thickness of the wafer (525 µm).

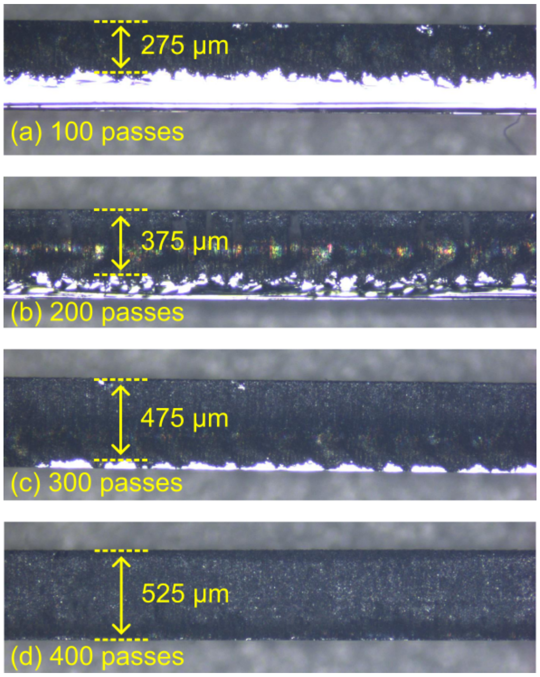

Figure S2. Microscope images of the edge of the die after (a) 100, (b) 200, (c) 300, and (d) 400 passes of the dicing laser (scribed surface up). Measured scribe depths of $\approx$ 52, 71, 90, and 100 % of total thickness (525 μm) are indicated, respectively, increasing linearly with passes.

An optimal number of 300 passes was chosen to dice samples for this study, with the front side affixed to dicing tape, as cutting 90 % through the wafer from the backside protects the surface of interest and keeps the individual die attached to one another during the process, yet still allows for easy separation maximising yield.

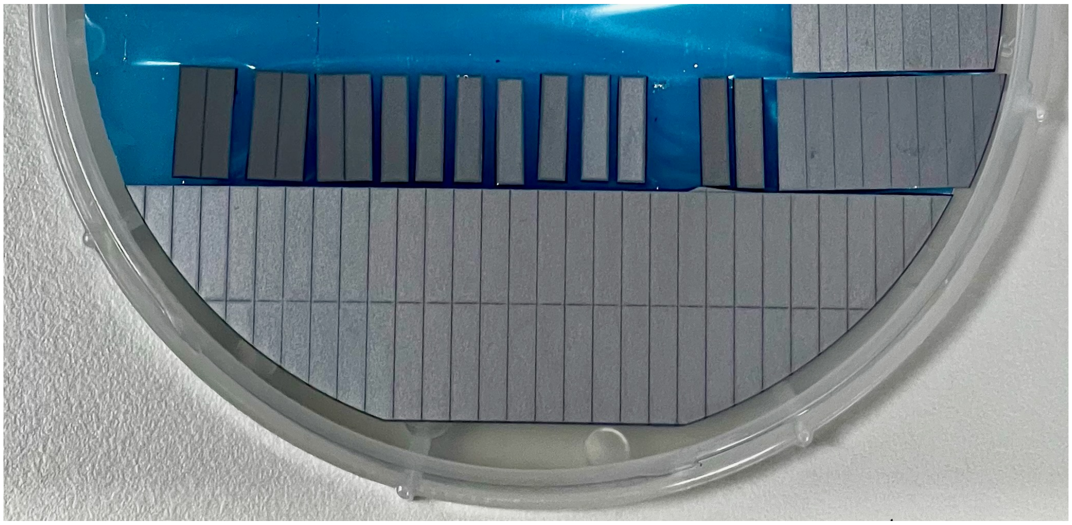

Figure S3. The backside of a silicon wafer mounted on dicing tape which has been scribed in a 3 x 12 mm grid with 300 passes. Some die have been separated to demonstrate the yield, repeatability of dimensions, and ease of separation.

Transmission spectroscopy

The broadband light source used for these measurements is an Ocean Optics DH-2000-BAL deuterium tungsten halogen Light Source. The detecting spectrometer is an Ocean Optics FLAME-T-UV-VIS-ES linear silicon CCD array. Ocean Optics QP450-1-XSR stainless steel shielded optical fibres transmit light from the source through a collimating lens, and collect transmitted light to the spectrometer through a large aperture collector lens [Figure S4].

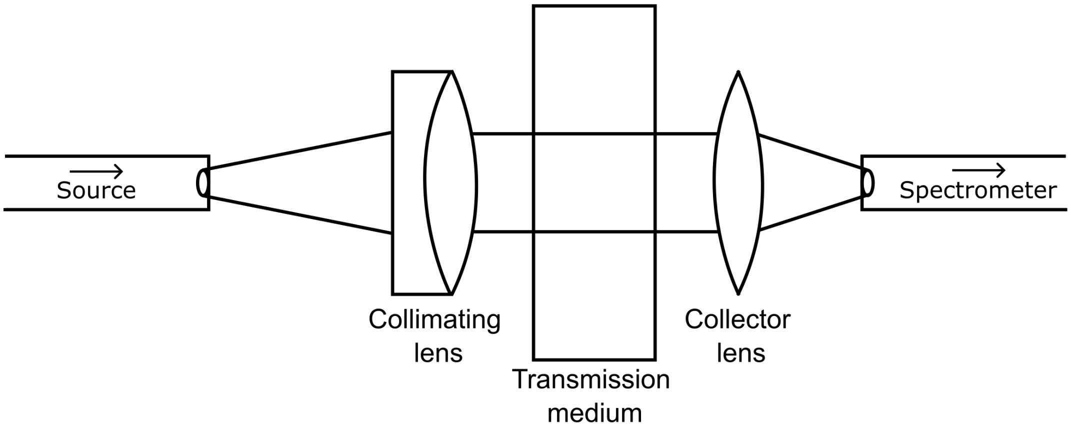

Figure S4. Schematic of the set-up used to measure the transmittance of the pressure cell window and precursor gasses.

Dark measurements represent the measurement of background intensity of the lab with the spectroscopy light source off. Source measurements are taken with no transmission medium (only an air gap) between the source and collector fibres. The “window” spectrum is that of the light transmitted through the pressure cell window [Figure S5 (a)]. Window transmittance is calculated as the percentage of the source spectrum that is transmitted through the pressure cell window [Figure S5 (b)].

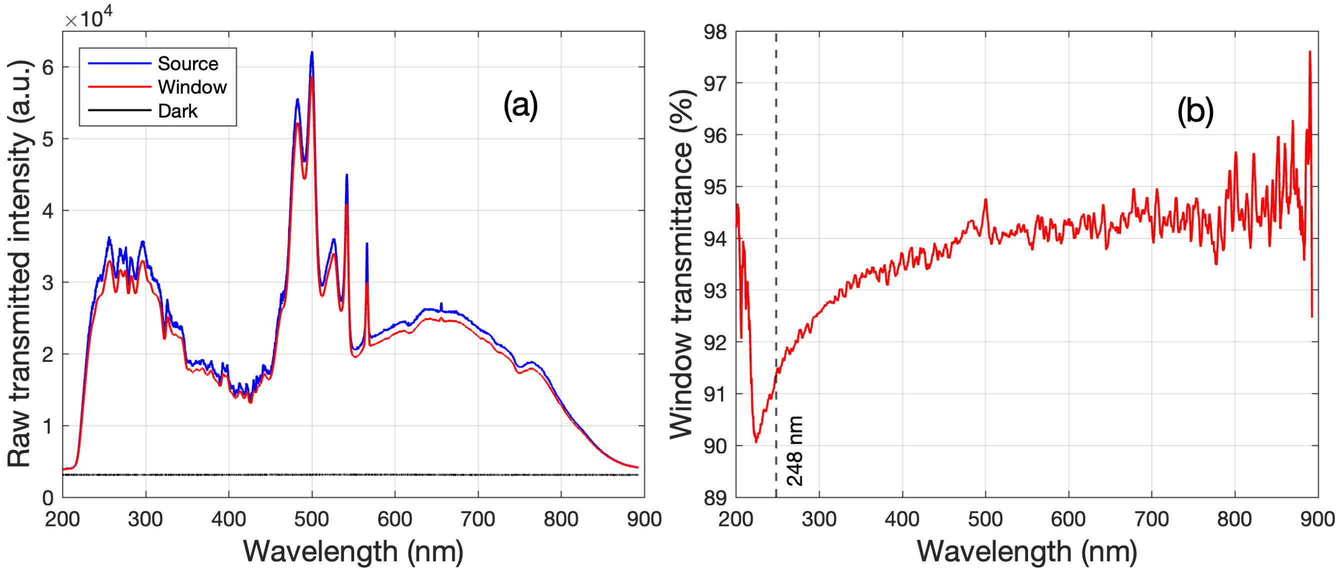

Figure S5. (a) Raw transmitted intensity of source, fused silica pressure cell window, and dark background. (b) Transmittance of the pressure cell window, measured to be >90 % at 248 nm.

To measure the transmittance of the high-pressure gas environments used for this study, a dual-window pressure was constructed [Figure S6. The cell is then filled with a gas of interest, and the transmittance is calculated as the percentage of the spectrum that is transmitted through the gas being measured compared to the cell filled with air of the same pressure [Figure S7].

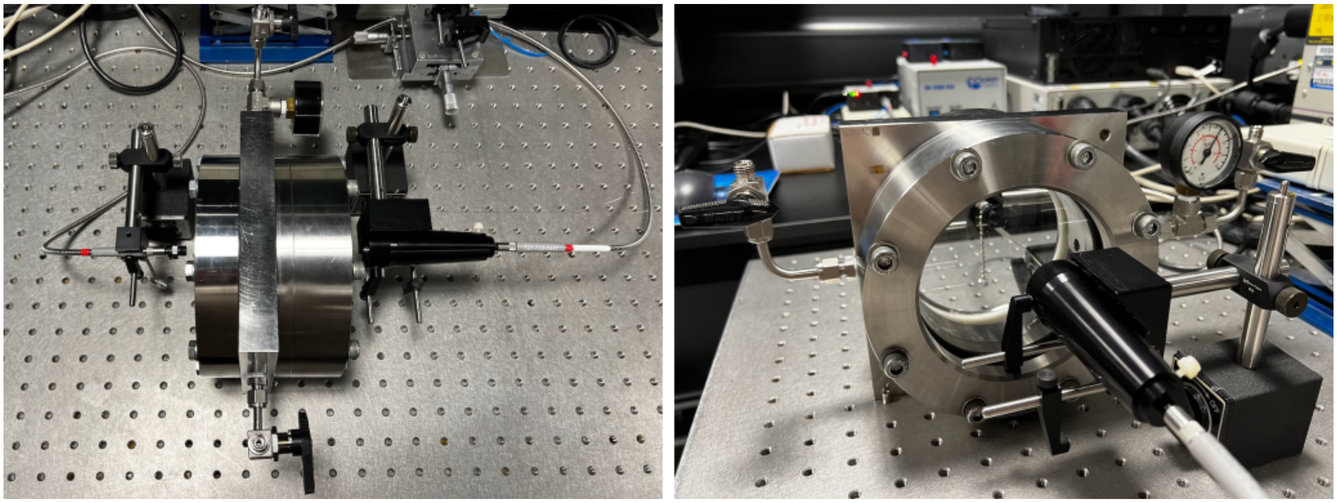

Figure S6. The dual-window pressure cell used to measure the transmittance of the precursor gasses.

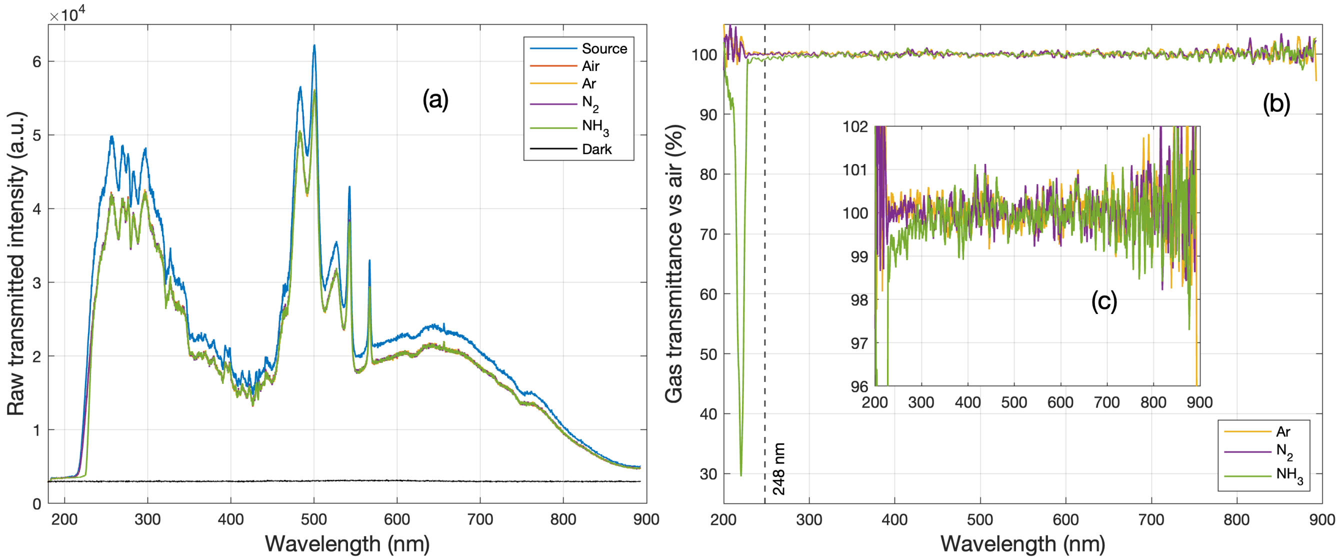

Figure S7. (a) Raw transmitted intensity of the source (no pressure cell), and the gasses (contained within a dual-window pressure cell): Air, Ar, N_2, NH_3, and dark background. (b) The transmittance of the gas environments used in this study shows all precursor gasses to have a 248 nm transmittance of 100 ± 1 % compared to air. We observe NH_3 having a strong UV absorption at ≈ 220 nm. (c) Inset restricted to 96-102 % to demonstrate 100 % effective transmittance.

Excimer beam profiling

The spectral intensity profile of the excimer laser was measured using an Ocean Optics FLAME-T-UV-VIS-ES CCD spectrometer [Figure S8]. This confirms the 248 nm wavelength of the KrF stimulated emission, and its spectral line width broadening.

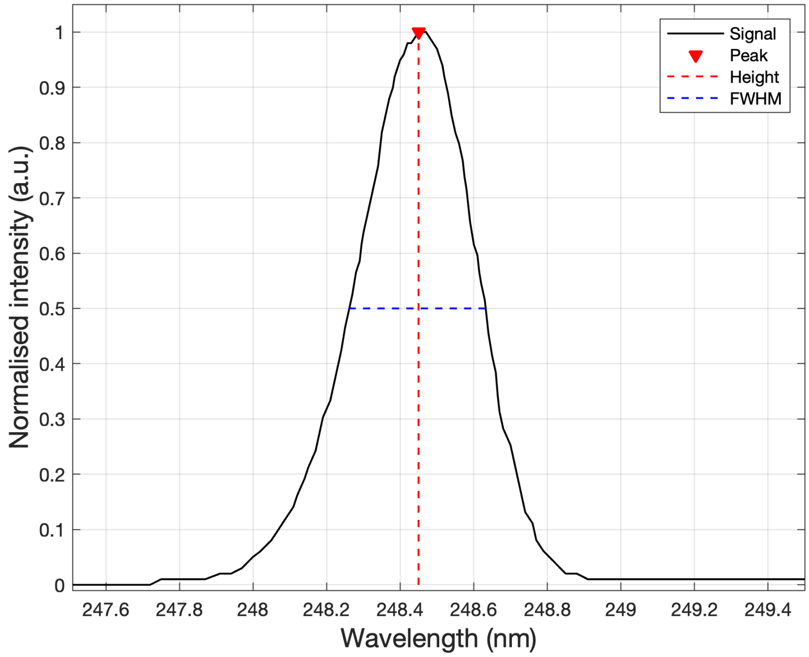

Figure S8. Normalised spectral intensity profile of excimer pulse. Peak wavelength = 248.45 nm with a FWHM broadening of ≈ 370 pm.

Temporal intensity profile measurements were taken using a Thorlabs DET025A/M PIN fast photodiode and quantify the 42 ns pulse duration of the excimer beam [Figure S9].

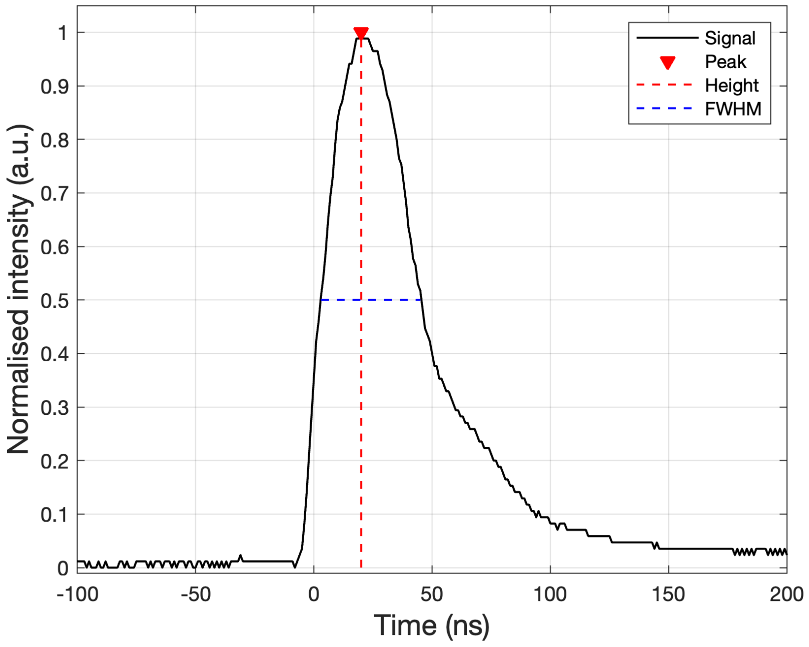

Figure S9. Normalised temporal intensity profile of a 2.8 J/cm^2 excimer pulse. FWHM pulse width = 42 $\pm$ 2 ns.

A Gentec-EO Beamage-4M CCD spatial intensity profiler with a BSF23G11.3N UV-Vis fluorescent crystal converter was used to determine the lateral dimensions of 2.5 x 2.5 mm^2 and quantify the homogenisation performance of the focused excimer spot incident on the Si surface.

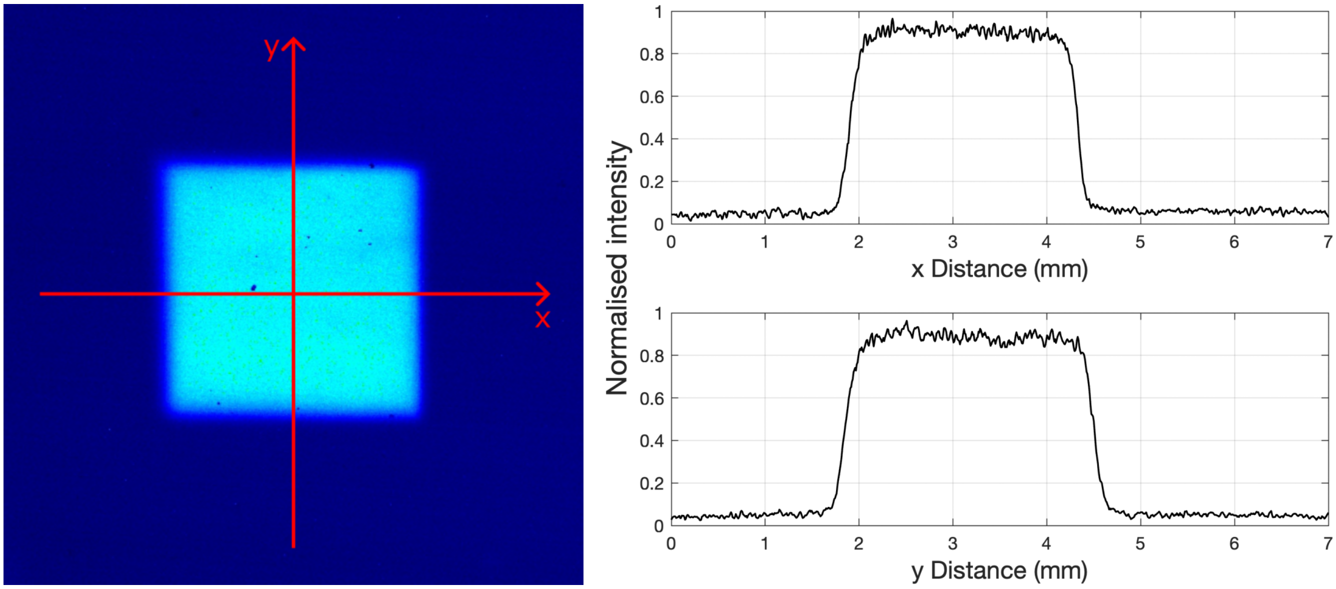

Figure S10. Left: Beam profiler image of excimer spot. Right: Normalised spatial intensity x and y line profiles taken through the centre of the spot.

Optical properties of silicon

Wavelength-dependent optical constants of Si taken from J. A. Woollam's SI_JAW3 material file on their IR VASE II ellipsometer. This file was generated from their work with Herzinger et al on the study "Ellipsometric determination of optical constants for silicon and thermally grown silicon dioxide via a multi-sample, multi-wavelength, multi-angle investigation".[38]

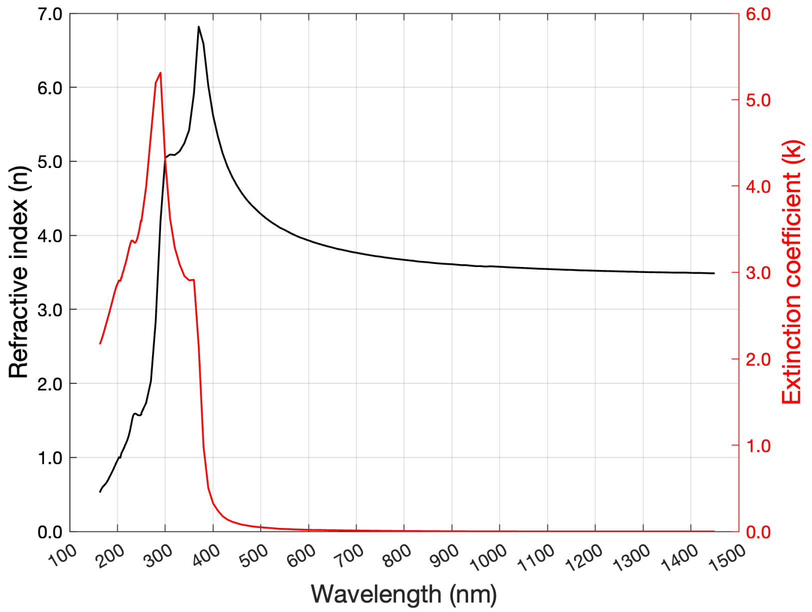

Figure S11. Refractive index (n) and extinction coefficient (k) of Si.

The absorption coefficient (α) and penetration depth (δ_p) of Si are calculated as a function of wavelength using the following relations: $\alpha = 4\pi k/\lambda$ and $\delta_p = 1/\alpha$, respectively. Also displayed is the absorption edge of Si, determined by its band gap (E_g) of 1.12 eV at 300 K, corresponding to $\lambda = hc/E_g$ = 1107 nm.

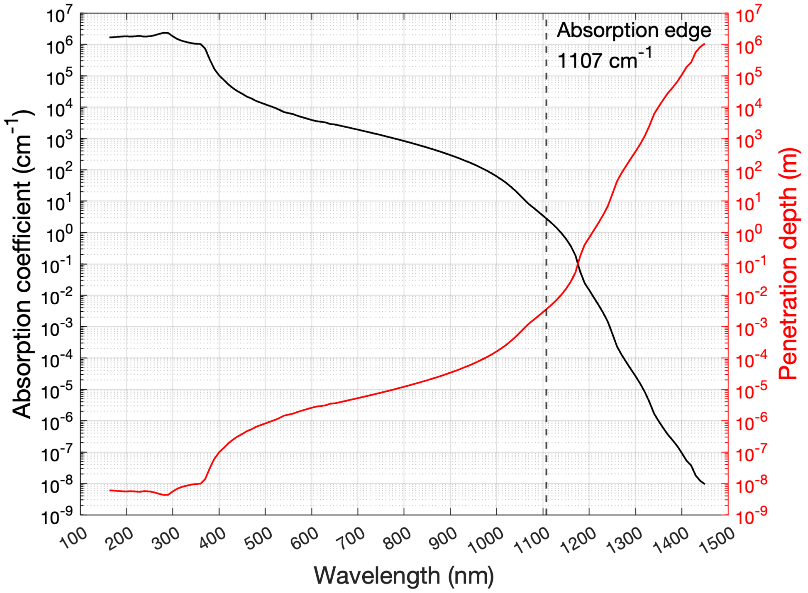

Figure S12. Absorption coefficient (α) and penetration depth (δ_p) of Si, calculated from the optical constants n, k as a function of wavelength.

Optothermal simulations methodology

We employ a specialised simulation tool on optothermal modelling to assist in gaining insights into the laser annealing of intrinsic Si. Specifically, we employ strongly coupled optical and thermal simulations considering the optical and thermal properties of Si including their temperature-dependent behaviour, and annealing. In particular, we utilise a 1D optical transfer matrix calculation and 1D time evolution of heat and combine the two into a strongly coupled self-consistent module, that contains all the material properties one would require, including optical dispersion and thermal properties (heat capacity, thermal conductivity) that are temperature dependent and phase transformation of materials (i.e., explicitly include melting point and latent heat of phase transformation). In the following section, we describe the main physics and physical models solved as well as the methodology. Optical simulation: The optical component of laser annealing is solved in 1D under normal incidence

within the frame of Fresnel equations using the transfer matrix method[51,52]. Specifically, the so-called "propagation" and "interface" transfer matrices are formulated based on the propagation properties and boundary conditions of the electromagnetic fields of the two counter-propagating waves within the structure, at the material's bulk and interfaces, respectively. Then the total optical problem at a specific time instance is solved by just multiplying the respective transfer matrices, starting from the incident side (unitary incidence and reflected amplitude, r) all the way to the back side of the device (transmitted amplitude, t)

$$\begin{pmatrix} t \\ 0 \end{pmatrix} = M \begin{pmatrix} 1 \\ r \end{pmatrix} \tag{S3}$$

where the transfer matrix M is obtained as a product of propagation matrices P_i and interface matrices $\mathrm{I}_{i,i+1}$

$$M = I_{n-1,n}P_{n-1} \ldots\ I_{2,3}P_2I_{1,2} \tag{S4}$$

with the index running over all layers in the multilayer structure. The individual matrices are

$$I_{i,i+1} = \frac{1}{2}\begin{pmatrix} 1+n_i/n_{i+1} & 1-n_i/n_{i+1} \\ 1-n_i/n_{i+1} & 1+n_i/n_{i+1} \end{pmatrix}, \quad P_i = \begin{pmatrix} e^{i2\pi n_i d_i/\lambda} & 0 \\ 0 & e^{-i2\pi n_i d_i/\lambda} \end{pmatrix} \tag{S5}$$

The solution of Equation S3 yields the reflection and transmission amplitudes, r and t. Also, the net electromagnetic flux, $I(x)$, in each point of the structure can be calculated by

$$I = Re\{n\}\left(\left|E_f\right|^2 - |E_b|^2\right) - 2Im\{n\}\,Im\{E_f E_b^*\} \tag{S6}$$

where E_f and E_b the forward and backward propagating waves at point x and n is the complex refractive index. The absorption within a Δx element is then $A(x) = (\partial I/\partial x)\,\Delta x$. This absorption calculation is done at every point in the device based on the discretisation Δx we have assumed (in our case 5 nm per grid), on which the heat transport simulation will be performed. Coupled with the spectrum of the laser [Figure S8] we get a spectral absorption profile, which when integrated over all wavelengths yields the total absorption distribution in the whole structure for unit power incidence. Finally, when this is coupled with the temporal envelope of the laser [Figure S9] it becomes the distributed time-dependent heating source to be used in the heat diffusion simulations. The refractive index of Si is assumed to be temperature-independent. Thermal simulation: The heat diffusion equation in 1D is solved on the above-mentioned grid

$$c_p\rho\frac{\partial T}{\partial t} = \frac{\partial}{\partial t}\left(k\frac{\partial}{\partial x}\right) + \dot{q} \tag{S7}$$

where c_p is the specific heat capacity (in J/KgK), ρ is the mass density (in Kg/m^3), k is the thermal conductivity (in W/mK), and $\dot{q}$ is the time-dependent thermal load coming from light absorption, as explained in the optical section, above. All the above quantities are a function of space (different materials) and temperature (nonlinear response). Equation S7 is solved using the 4th-order Runge-Kutta integration scheme. Boundary conditions are implemented on both sides including thermal insulation, fixed temperature, natural or forced convection, and thermal radiation. Material nonlinearities include: Heat capacity temperature dependence, described following the relationship

$$c_p(T) = a + b\frac{\left(\frac{T}{300}\right)^c - 1}{\left(\frac{T}{300}\right)^c + b/a} \tag{S8}$$

where $a,\ b,\ c$ are adjustable parameters fitter to experimental results. For Si we extract $a = 708$, $b = 180$, $c = 1.99$. Figure S13 shows the heat capacity model fit to Si, used in our solver. Thermal conductivity temperature dependence is described by the following relation

$$k(T) = a\left(\frac{T}{300}\right)^b + c \tag{S9}$$

where $a,\ b,\ c$ are adjustable parameters fitter to experimental results. For Si we extract $a = 130$, $b = -1.6$, $c = 12$. Figure S14 shows the thermal conductivity model fit to Si, used in our solver. Please note that our model is valid for temperatures above RT, which is valid for our experimental case. The latent heat of melting/solidification assumes a narrow Gaussian in the heat capacity, whose integral is equal to the heat of fusion.

$$c_p^f(T) = \frac{a}{b\sqrt{\pi}}e^{-(T-T_c)^2/b^2} \tag{S10}$$

where a is the specific heat of fusion (in J/Kg), T_c is the melting point (in K) and b is a nonzero bandwidth (in K), typically around 10 K, to allow smooth evaluation of temperature derivatives. Figure S13 Heat capacity temperature dependence: blue dots are the experimental data retrieved from[53] and the red solid line is the fitting curve. Figure S13 Thermal conductivity temperature dependence: blue dots are the experimental data retrieved from[54] and the red solid line is the fitting curve. The simulated peak melt depth and total melt duration are calculated by isolating the isoline at T_m = 1687 K [Figure 5]. For a pulse of 1.5 J/cm^2, peak melt depth = 296 nm reached at 55 ns, total melt duration = 116.5 ns. For a pulse of 2.8 J/cm^2 pulse, peak melt depth = 812 nm reached at 109 ns, total melt duration = 395.5 ns. These values of melt duration are in excellent agreement with those measured by TRR of 116 and 397 ± 2 ns, respectively.

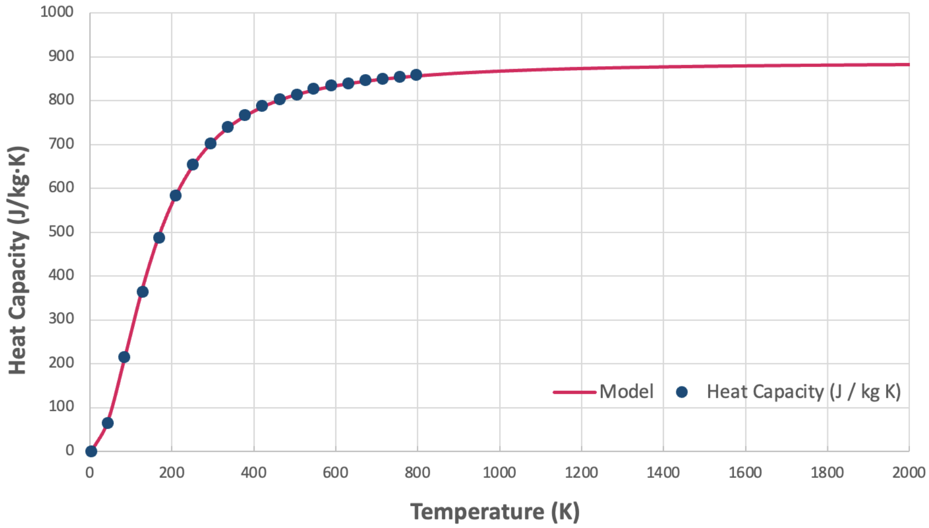

Figure S13. Heat capacity temperature dependence. Blue data points are the experimental data retrieved from Marchbanks et al[53] and the pink solid line is the fitting curve.

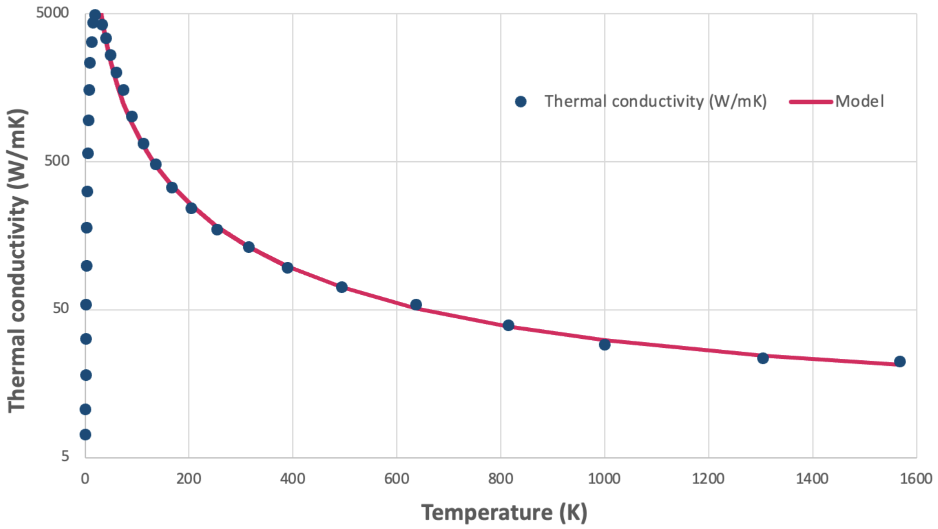

Figure S14. Thermal conductivity temperature dependence. Blue data points are the experimental data retrieved from Ziabari et al[54] and the pink solid line is the fitting curve.

TRR

Excimer pulses were measured across fluences of 1.5 - 3.0 J/cm^2 [Figure S15 (a)]. These display the repeatability of intensity output over many pulses. Taking the sum intensity within the same time range shows the linear intensity response of the photodiode (b). Normalising the signals shows the consistency of the pulse profile and duration (c). The standard deviation gives an average pulse-to-pulse intensity variation of only ≈ 1.2 % (d).

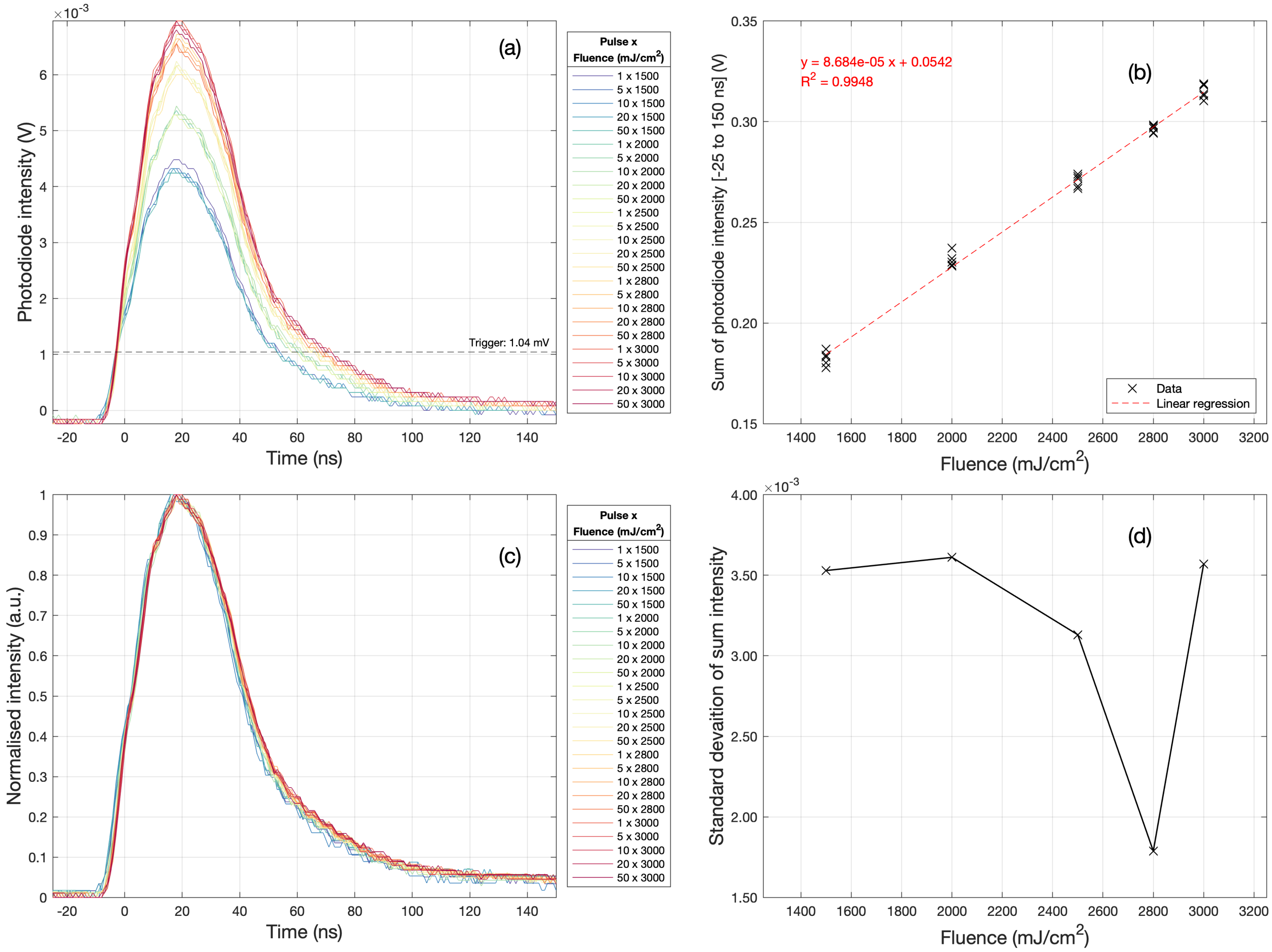

Figure S15. (a) Measured photodiode voltage intensity of 1-50 excimer pulses as a function of time for fluences of 1.5 to 3.0 J/cm^2. (b) The sum of photodiode voltage in the time range -25 to 150 ns as a function of fluence, displaying a linear response to incident intensity. (c) Normalised intensity for all pulses, displaying the consistency of the temporal pulse profile across all fluences and pulses. (d) The standard deviation of the summed intensity at each fluence in (b).

To correlate V_{pd} to R_{Si}, we follow the methodology of Diez et al[35]. Where A is a measure of photodiode responsitivity, and B accounts for signal offset, V_{pd} can be expressed by

$$V_{pd} = A \times I_{refl} + B = A \times (I_{probe} \times R_{Si}(T)) + B \tag{S11}$$

Before the excimer pulse, the average constant probe voltage measured by the photodiode, $\bar{X}(V_{pd,\,RT})$, corresponds to the known reflectivity of Si at RT, $R_{Si,\,RT} = 34.8\%$[55] at λ = 632.8 nm. A few ns after the excimer pulse, the probe signal plateaus at an approximately constant average maximum voltage, $\bar{X}(V_{pd,\,liq.})$, corresponding to the known reflectivity of liquid Si, $R_{Si,\,liq.} = 71.2\%$[56].

$$\bar{X}(V_{pd,\,RT}) = A \times 34.8\ \% \ + B \tag{S12}$$

$$\bar{X}(V_{pd,\,liq.}) = A \times 71.2\ \% \ + B \tag{S13}$$

By way of simultaneous equations, the values of A and B are determined, and in turn, we solve Equation S11 for R_{Si} to calculate the reflectivity of silicon from the measured voltage.

$$R_{Si} = \frac{V_{pd} - B}{A \times I_{probe}} \tag{S14}$$

For a single excimer pulse on virgin silicon, we observe the melt duration increase with fluence [Figure S16]. We also observe the onset of fixed-frequency oscillations in reflectance of ≈ 6.67 MHz across the maximum intensity of the probe signal for

fluences of 2.0 J/cm^2 and above. This is not seen for lower fluences at which melting is still observed. This effect is the result of "overheating" through electron-phonon coupling, where photoexcited carriers induce the generation of intense phonons which propagate into the samples, modulating the reflectivity.

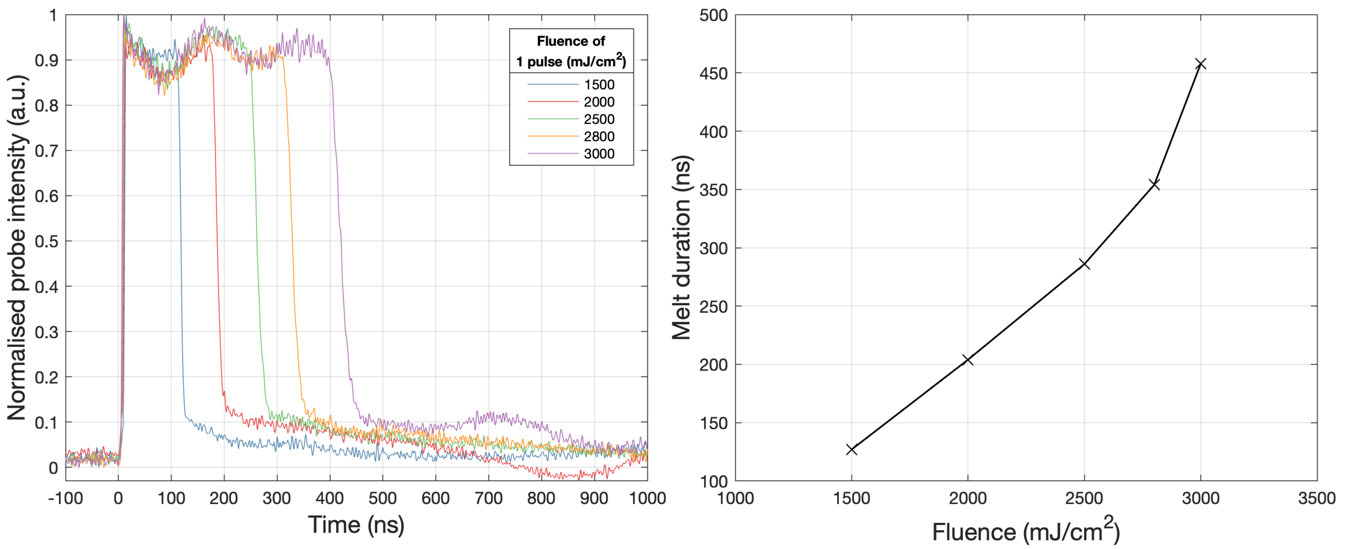

Figure S16. TRR signature of Si processed with 1 pulse of 1500, 2000, 2.5, 2.8, and 3.0 J/cm^2 in 10 bar N_2. We observe a lengthening of the melt duration with fluence and a very consistent, repeatable wave pattern in the signature across the maximum intensity of the melt.

For 1.5 J/cm^2 we observe a consistent TRR signature displaying initial heating, phase transition, melt duration, and resolidification for 1 - 50 pulses (pulses above 10 are not shown as they are identical) [Figure S17 (a)]. For fluences of 2.5 J/cm^2 and above we observe melt duration increasing with pulses (b). Notably, we observe the progression of the reflectivity oscillation pattern in the probe signal increasing pulses from 1 - 10. 3D plots at a viewpoint of 5 ° Azimuth and 70 ° Elevation are included to display the consistency of the melt for many pulses of 1.5 J/cm^2 (c), and increasing melt duration and progression of the repeatable wave pattern across the maximum intensity of the melt (d). Pulse-to-pulse, the observed reflectivity oscillation has a consistent frequency, thus, as the number of pulses is increased and the melt duration lengthens and the number of observed peaks increases. The peak-to-peak amplitude of the reflectivity oscillations increases significantly with pulses.

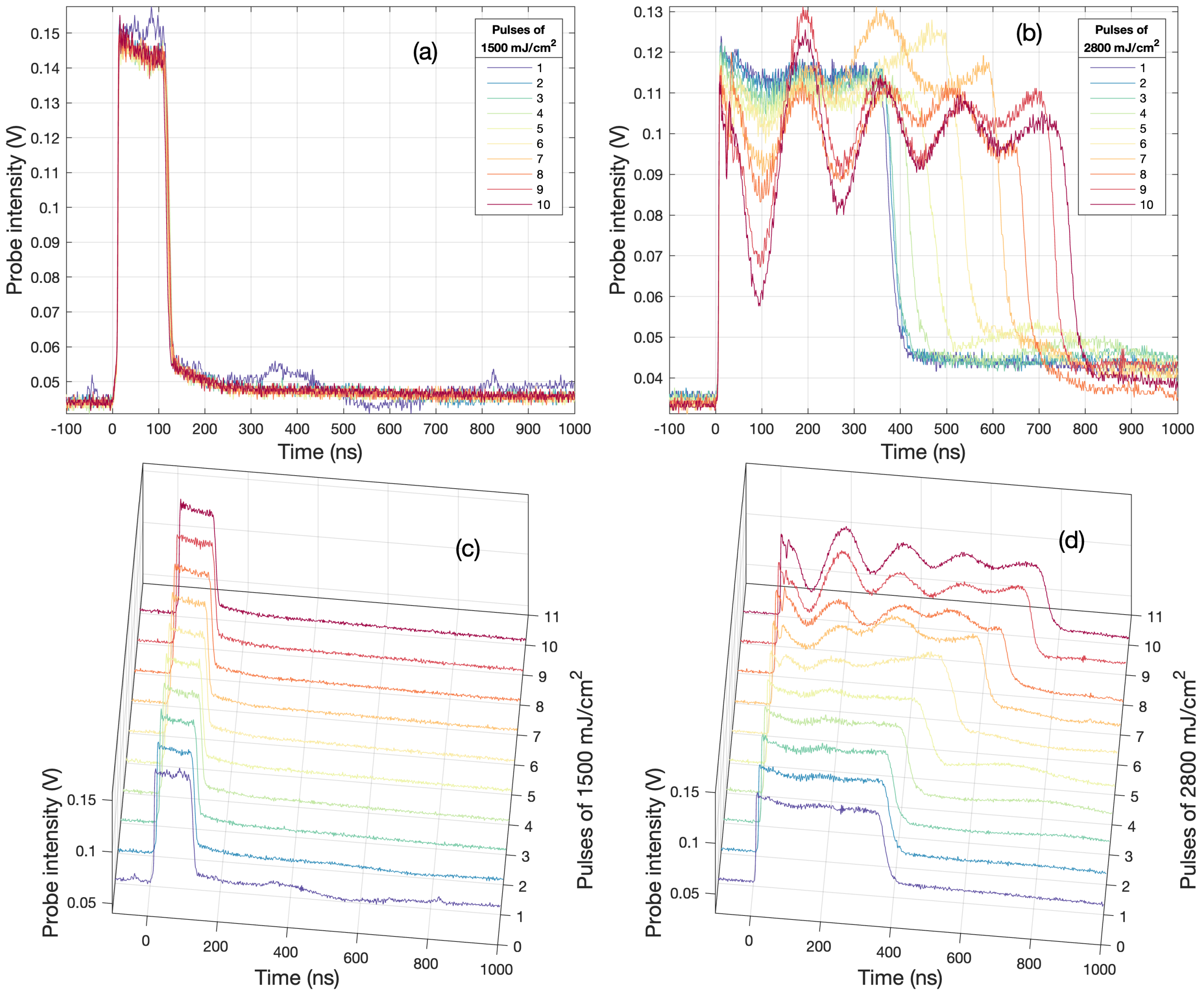

Figure S17. (a) Measured probe intensity of 1-10 pulses of 1.5 J/cm^2 in air. (b) Measured probe intensity of 1-10 pulses of 2.8 J/cm^2 in air. (c) 3D plot of (a) at a viewpoint of 5 ° Azimuth and 70 ° Elevation to emphasise the consistency between TRR signatures when compared to (d) 3D plot of (b) at a viewpoint of 5 ° Azimuth and 70 ° Elevation to display the increasing melt duration and development of the repeatable wave pattern across the maximum intensity of the melt.

EPR cavity signal

A low-temperature EPR measurement of the cavity, only containing an empty quartz sample tube, demonstrates that the broad dip observed between $\approx$ 320 - 340 mT is a feature of the equipment [Figure S18].

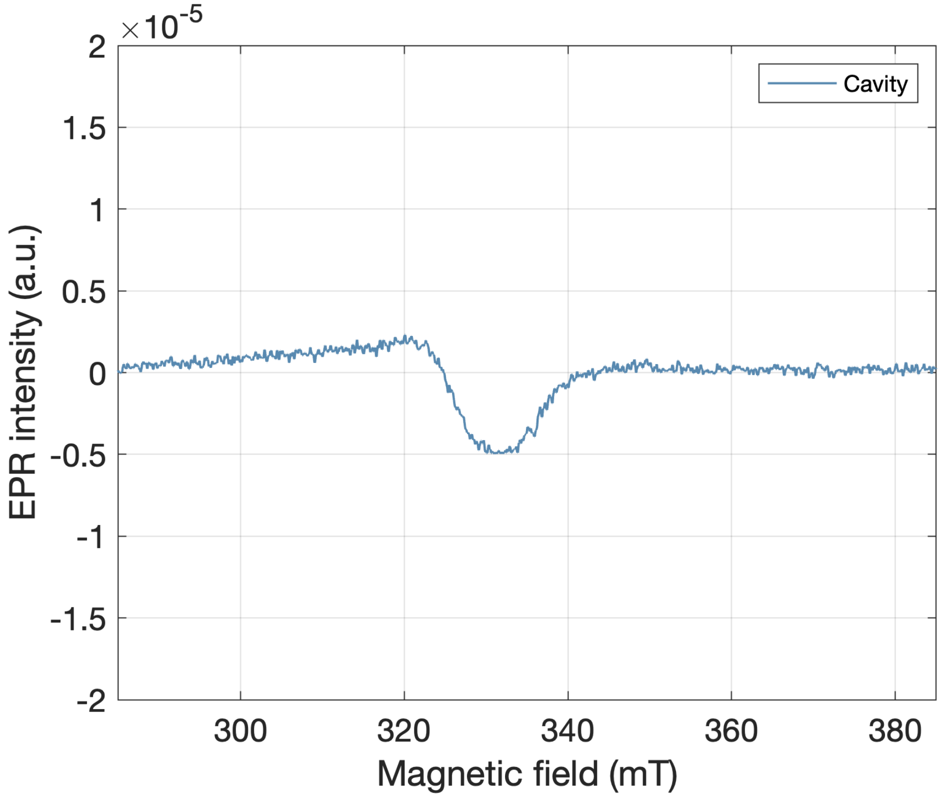

Figure S18. EPR measurement of the cavity only containing an empty quartz sample tube.

Microscope images of surface damage

An optical zoom camera was used to image a 2.5 x 2.5 mm^2 laser-irradiated spot of Si to observe the surface roughness as the result of damage from 50 pulses of 3.0 J/cm^2 [Figure S19]. A Microscope camera was used to take a closer image of the bottom left corner of the same damaged area showing undulations of varying size and shape [Figure S20]. Atomic force microscopy (AFM) was used to capture the topography of the surface undulations in greater detail [Figure S21]. All AFM images of surfaces damaged with fluences > 3.0 J/cm^2 display many narrow spikes [Figure S22], initially these were though to be artefacts or dirt on the cantilever. However, taking an scanning electron microscope (SEM) images of a damaged surface reveals these to be still be present [Figure S23 (a)]. Thus, we must conclude that spiking of the silicon surface results from laser processing. The surface undulations are visible in the greatest detail when not obfuscated by the spikes by flipping the 3D AFM image upside down [Figure S22 (b), rotated anti-clockwise by 180 °about the axis running from the bottom corner to the top corner, going up the page].

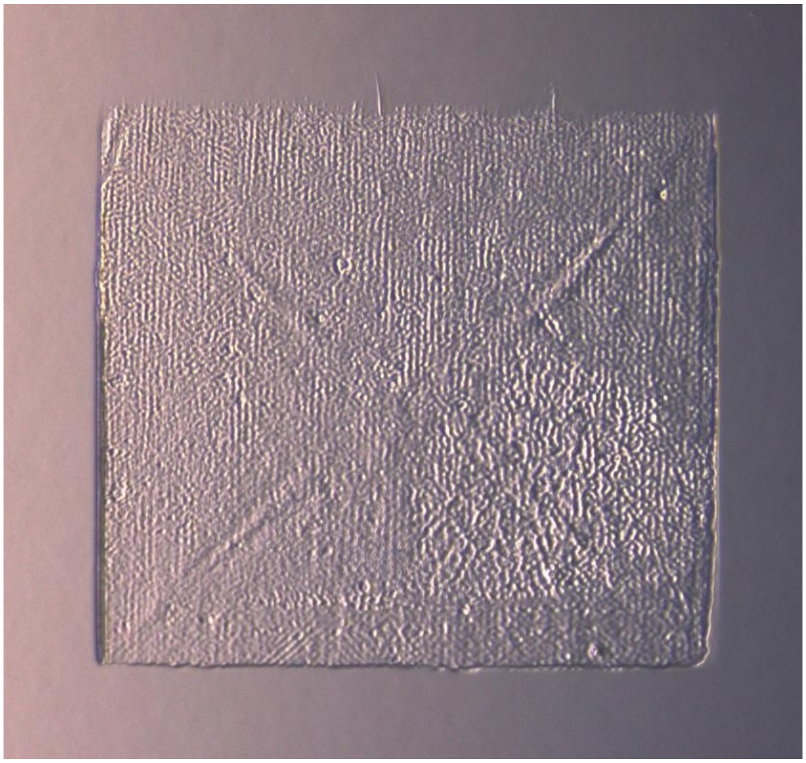

Figure S19. Optical zoom camera image of a 2.5 x 2.5 mm^2 laser-irradiated spot of Si displaying surface roughness as the result of damage from 50 pulses of 3.0 J/cm^2

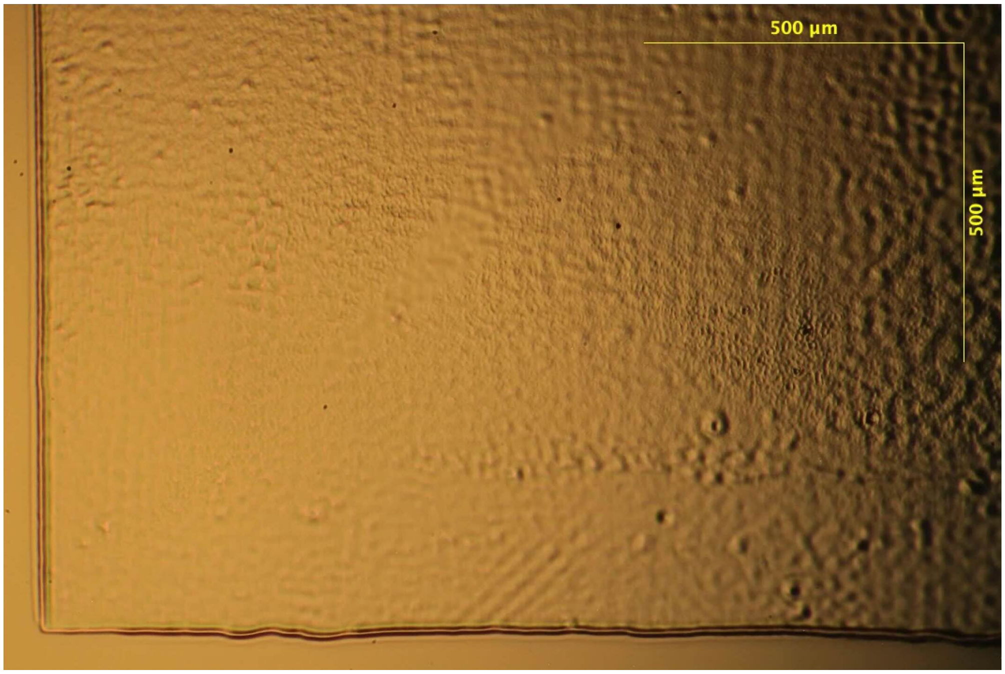

Figure S20. Optical microscope image of the lower left corner of a 2.5 x 2.5 mm^2 laser-irradiated spot of Si displaying surface undulations due to damage.

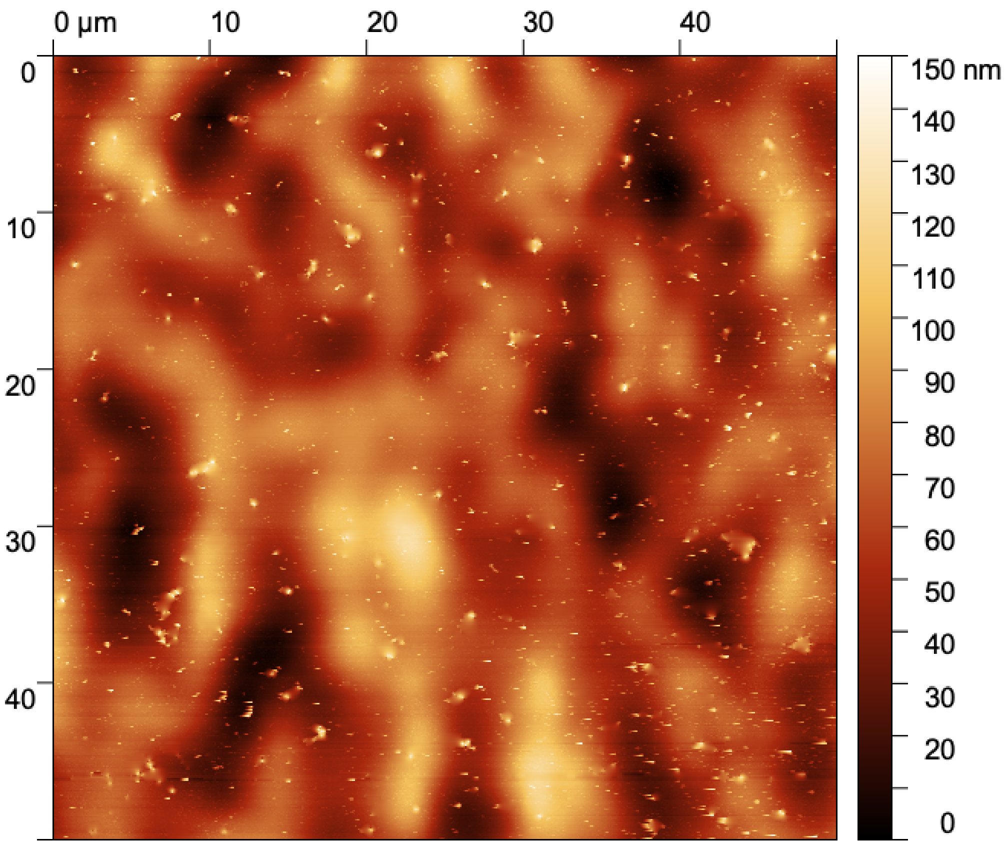

Figure S21. AFM image of silicon processed with 50 pulses of 3.0 J/cm^2 showing surface undulations due to damage and apparent spiking.

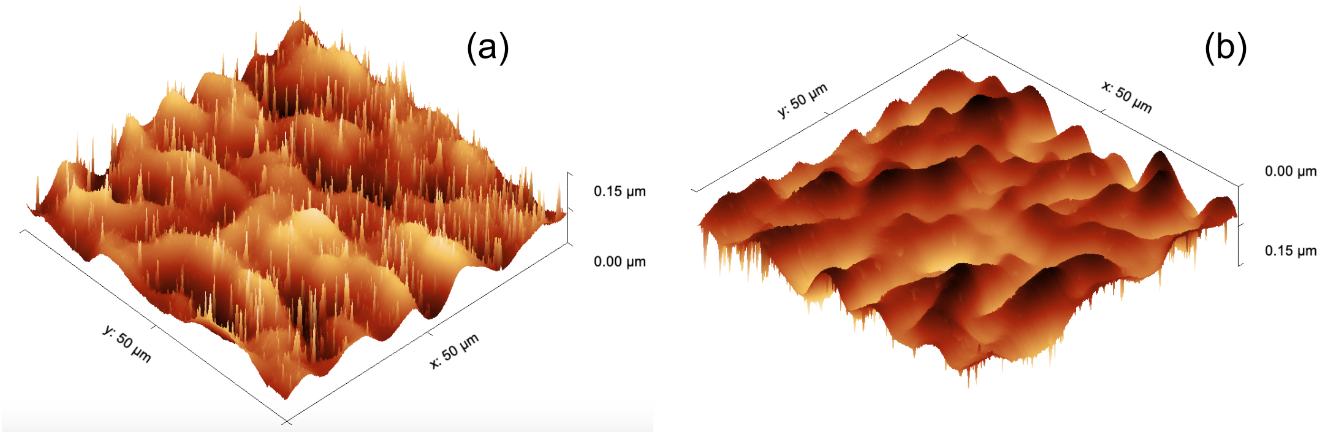

Figure S22. 3D AFM images of silicon processed with 50 pulses of 3.0 J/cm^2 showing (a) surface undulations and spiking (b) flipped upside down to view the topography of the undulations without being obfuscated by the spikes.

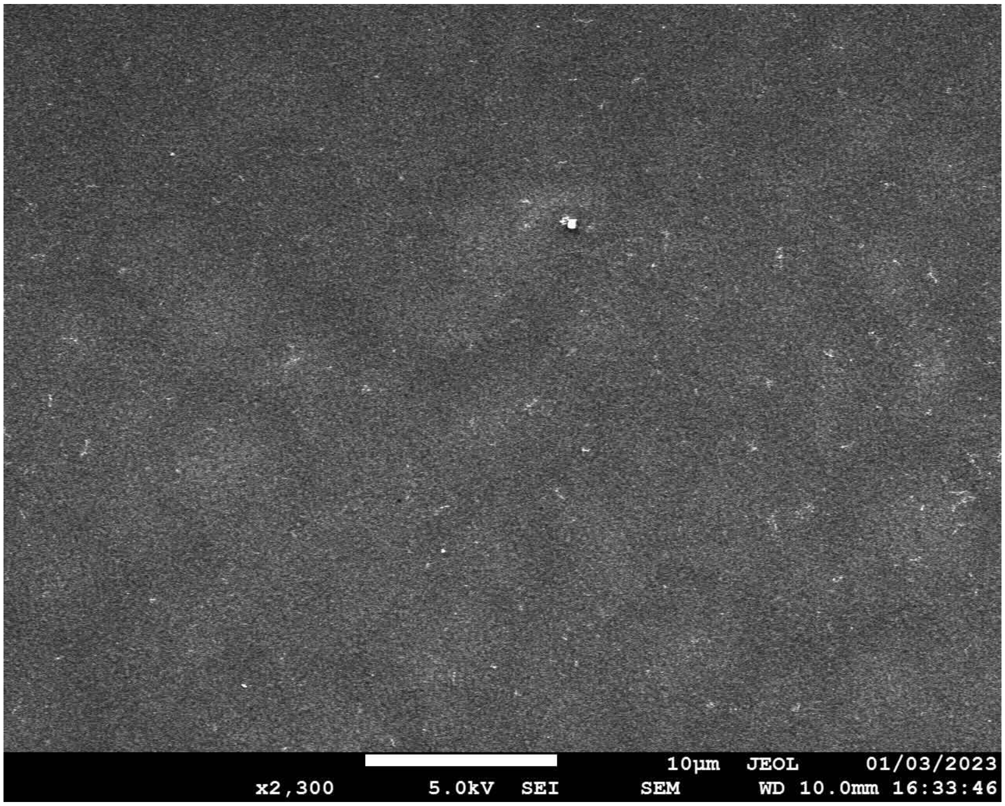

Figure S23. SEM image of silicon processed with 50 pulses of 3.0 J/cm^2 showing surface undulations due to damage and silicon spiking.

References (additional to the references in the main manuscript)

51. Yeh, P. Optical waves in layered media (John Wiley & Sons, 2005).

52. Lampadariou, E., Kaklamanis, K., Goustouridis, D., Raptis, I. & Lidorikis, E. Nonlocal Effective Medium (NLEM) for Quantitative Modelling of Nanoroughness in Spectroscopic Reflectance. Photonics 9, 499, DOI: 10.3390/photonics9070499 (2022).

53. Marchbanks, C. & Wu, Z. Reduction of heat capacity and phonon group velocity in silicon nanowires. J. Appl. Phys. 117, DOI: 10.1063/1.4913453 (2015).

54. Ziabari, A., Bian, Z. & Shakouri, A. Adaptive Power Blurring Techniques to Calculate IC Temperature Profile under Large Temperature Variations. Proc. Int. Microelectron. Assembly Packag. Soc. (2010).

55. Green, M. A. Self-consistent optical parameters of intrinsic silicon at 300K including temperature coefficients. Sol. Energy Mater. Sol. Cells 92, 1305–1310, DOI: 10.1016/j.solmat.2008.06.009 (2008).

56. Jellison, G. E. & Lowndes, D. H. Measurements of the optical properties of liquid silicon and germanium using nanosecond time-resolved ellipsometry. Appl. Phys. Lett. 51, 352–354, DOI: 10.1063/1.98438 (1987).
